# Seed-mediated vertical transmission of *Pantoea* core endophytes

**DOI:** 10.1093/ismejo/wraf192

**Published:** 2025-08-28

**Authors:** Irene Sanz-Puente, Santiago Redondo-Salvo, Gloria Torres-Cortés, María de Toro, Susana Fernandes, Andreas Börner, Óscar Lorenzo, Fernando de la Cruz, Marta Robledo

**Affiliations:** Instituto de Biomedicina y Biotecnología de Cantabria (IBBTEC), Universidad de Cantabria—Consejo Superior de Investigaciones Científicas (CSIC), Santander, Spain; Instituto de Biomedicina y Biotecnología de Cantabria (IBBTEC), Universidad de Cantabria—Consejo Superior de Investigaciones Científicas (CSIC), Santander, Spain; Biomar Microbial Technologies, Parque Tecnológico de León, Armunia, Lesón, Spain; Institut Agro, INRAE, IHS, Université d'Angers, Angers, France; Innoplant S.L, Avenida Alfaguara 62, Alfacar, Granada, Spain; Genomics and Bioinformatics Core Facility, Center for Biomedical Research of La Rioja, Logroño, Spain; Departamento de Botánica y Fisiología Vegetal, Instituto de Investigación en Agrobiotecnología (CIALE), Universidad de Salamanca, Salamanca, Spain; Genebank Department, Leibniz Institute of Plant Genetics and Crop Plant Research (IPK), Seeland/OT, Gatersleben, Germany; Departamento de Botánica y Fisiología Vegetal, Instituto de Investigación en Agrobiotecnología (CIALE), Universidad de Salamanca, Salamanca, Spain; Instituto de Biomedicina y Biotecnología de Cantabria (IBBTEC), Universidad de Cantabria—Consejo Superior de Investigaciones Científicas (CSIC), Santander, Spain; Instituto de Biomedicina y Biotecnología de Cantabria (IBBTEC), Universidad de Cantabria—Consejo Superior de Investigaciones Científicas (CSIC), Santander, Spain; Biomar Microbial Technologies, Parque Tecnológico de León, Armunia, Lesón, Spain

**Keywords:** plant-associated microorganisms, endophytes, seeds, vertical transmission, wheat, bacteria-host coevolution, core microbiota

## Abstract

Plant-associated microorganisms, particularly endophytes, are essential for plant health and development. Endophytic microbiota is intimately associated with host plants colonizing various tissues, including seeds. Seed endophytes are particularly noteworthy because of their potential for vertical transmission. This pathway may play a role in the long-term establishment and evolution of stable bacteria-host interactions across plant generations. Hundreds of seed-bacteria associations have been recently uncovered; however, most seem to be transient or unspecific. Although it is known that microorganisms can be transmitted from plant tissues to seeds and from seeds to seedlings, the experimental confirmation of bacterial transfer through successive plant generations by inoculation remains unreported. In this study, we identified *Pantoea* as the unique core endophytic bacteria inhabiting the endosperms of 24 wheat seed samples originally harvested in different worldwide locations. *Pantoea* is the genus with the highest relative average abundance in wheat seeds (61%) and in germinated roots and shoots grown under gnotobiotic conditions (45–38%). In the field, it was the only genus dwelling roots, shoots, spikes, and seeds of four different wheat varieties tested and its abundance progressively increased across these tissues. This genuine pattern of vertical enrichment, which was not found in other common wheat-associated taxa, suggests a role in the transfer of these endophytic bacteria through the seeds. To confirm intergenerational transmission, parental plants were inoculated with labelled *Pantoea* isolates, which specifically colonized the next generations of *Poaceae* plants, experimentally demonstrating bacterial vertical inheritance to the offspring generations and suggesting transmission specificity.

## Graphical Abstract

**Graphical Abstract:**
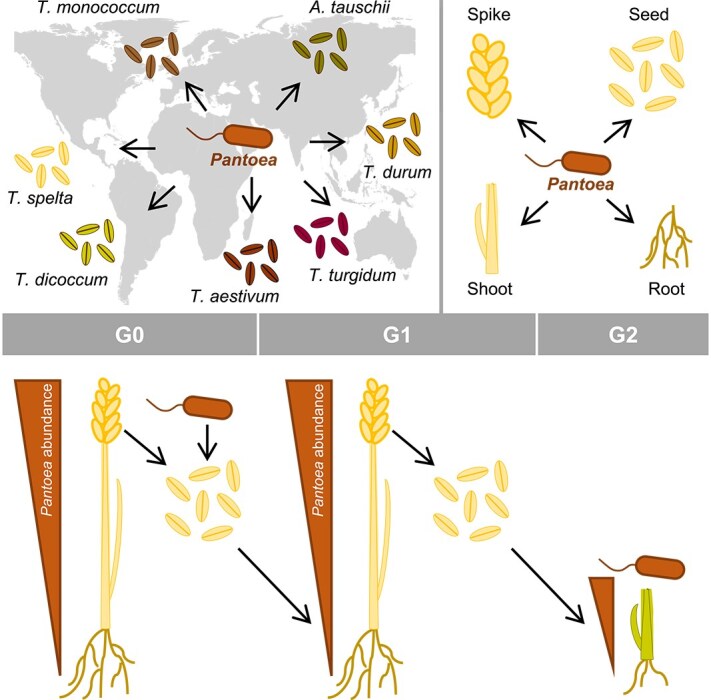

## Introduction

All multicellular organisms host complex microbial communities [1]. In humans, the maternal microbiome helps establish progeny intestinal flora, immune system, and metabolism [2]. Plants also host diverse microbial communities, forming the “holobiont” [3]. A healthy plant holobiont fosters plant-microbial homeostasis [4, 5]. Microorganisms that colonize internal plant tissues without causing apparent damage (i.e. endophytes) are critical for plant health [6, 7]. One well studied example is the nitrogen-fixing bacteria *Rhizobium leguminosarum,* which can survive in the soil or internally colonize legume root nodules to fix nitrogen [8]. Certain rhizobial strains can also colonize cereal or vegetables as growth-promoting endophytes [9, 10].

Research traditionally focused on rhizosphere and phyllosphere microbiota communities [3]. However, the microbiota associated with reproductive organs is recently receiving more attention [11–14]. Seeds, essential for plant regeneration, were long considered axenic [15, 16]. However, seed tissues harbor complex microbial communities, which may exert beneficial or deleterious effects on plant growth and health [4, 17]. In this research direction, recent studies have identified the presence of bacteria within seed structures [18, 19]. Although the internal colonization of seed-borne pathogens has been well established [20], it is only more recently that similar investigations have extended to putatively non-pathogenic, potentially mutualistic microbes.

Three major transmission pathways of seed-borne microorganisms have been suggested: external (surface contact), floral (through the stigma), and internal (via the vascular system) [12, 17, 21]. The external pathway is considered the most permissive route, whereas internal transmission is probably more restricted to endophytes [12, 22]. Microscopic studies have recently confirmed the presence of vertically-transmitted *Burkholderia* in the flower buds, close to the embryos, but not in the vascular tissues [23]. However, *Xanthomonas* was observed in connections of maternal vascular tissues to seeds and in the embryo [24, 25]. Microbial seeds-to-seedling transmission is well studied [26], particularly in relation to pathogens [20, 27]. Amplicon sequencing has suggested transmission of certain taxa across plant generations [28–31]. Several works isolating the same bacterial species from both G0 and G1 seeds also indicate natural transmission [28, 32, 33]. A study carrying out microbiota sequencing analysis of seeds over several generations suggests that only few endophytes might be consistently transmitted [28]. However, overlaps among microbiota members across tissues is not sufficient to experimentally prove vertical transmission and strain-specific identification is required to confirm seed-to-seed transmission.

Confirming vertical transmission is technically challenging. Several studies have verified that labelled GPF- or GUS bacteria provide a reliable approach for tracking endophytes seed dynamics [24]. Fruit and flower inoculation of GUS-labelled *Paraburkholderia phytofirmans* PsJN, isolated from onion, demonstrated endophytic colonization of the next generation in grapevines and maize [34, 35]. *Arabidopsis* roots inoculated with GFP-labelled *Bacillus thuringiensis* yielded bacteria in seedlings [36]. However, to the best of our knowledge, vertical transmission of native seed endophytes through several plant generations has not been confirmed using inoculation experiments.

This study tests the hypothesis that core bacterial endophytes in seeds are transmitted vertically across generations. To this end, we first profiled the seed endophytic communities of commercial and ancestral wheat (*Triticum*), one of the most cultivated cereals worldwide [37]. Sequencing analysis revealed that wheat seed endophytic microbiota from diverse geographic origins is dominated by *Pantoea*. To identify potential signs of vertical transmission, we tracked wheat prevalent taxa among different species and tissues. *Pantoea* dominance is maintained upon seedling germination in axenic conditions, but wild plants exhibit a distinct gradient across tissues, that culminates in seed recolonization. To investigate vertical inheritance, we finally set up a 3-year field experiment and established a model system based on GUS-labelled *Pantoea* that enabled us to document its persistence and inheritance through consecutive generations. Together, our combined sequencing surveys and multi-generational trials provide direct experimental evidence that bacteria can be vertically transmitted in plants.

## Materials and methods

A more detailed description of the methods is provided in the supplementary material.

### Plant material and soil sampling

The endophytic bacterial community of wheat was studied using 24 seed samples from two main sources (Tables S1–S3). One set of seed and plant material was collected from field plots in Spain at the end of the growing season. Rhizospheric soil surrounding wheat roots was also sampled. In the laboratory, plant samples were separated into root, shoot, spike, and mature seeds harvested from the spikes.

An additional set of seeds samples (Table S2), collected from various global locations, was regenerated at the IPK germplasm bank (Gatersleben, Germany). All samples were stored in paper bags at 4°C until processing.

For greenhouse experiments, seeds from *Triticum aestivum* (Tae_SP4; Table S3), *Lolium multiflorum*, and *Arabidopsis thaliana* Col-0 were used.

### Plant surface disinfection, bacterial isolation and DNA extraction

Plant tissues (~0.5 g) were immersed in 1 ml of Phosphate-buffered saline (PBS) containing 0.05% Tween-20 and sonicated for 1 min (Ultrasons, Barcelona, Spain). Three surface disinfection methods were tested [7, 25, 34], with the third method adopted for routine use. In this protocol, seeds, spikes, shoots, and roots were rinsed in 70% ethanol for 3 min (5 min for roots), followed by treatment with 5% active chlorine for 10 min (5 min for seeds). Samples were then rinsed three times with sterile distilled water. To confirm successful disinfection, 100 μl of the final wash rinse was plated on Tryptic Soy Agar (TSA; Condalab, Madrid, Spain) and incubated at 30°C for 3 days.

For endophytic bacterial isolation, ~0.25 g of surface-disinfected plant material was mechanically disrupted and incubated overnight in 1 ml PBS at 4°C. Serial dilutions of the suspension were plated on TSA and incubated at 30°C for at least 1 week.

DNA extraction from plant tissues for bacterial community analysis was performed using the DNeasy PowerLyser PowerSoil Kit (Qiagen, Hilden, Germany), following manufacturer’s instructions. DNA was eluted in 50 μl purified water and quantified using Nanodrop (ThermoFisher, Massachusetts, United States).

### Microbial communities sequencing and analysis

The V4 region of the 16S rRNA gene was amplified using primers 515_F and 808_R with Illumina overhang adapters (Table S4). PCR reactions contained 0.5 μM of each primer, 200 μM dNTPs, 0.02 U/μl Kapa2G Robust polymerase, and 25 ng of the template DNA. PCR amplification included an initial denaturation 98°C (30 s), 25 cycles (95°C, 15 s; 55°C, 15 s; 72°C, 10 s), and a final extension (72°C, 5 min). Amplicons were purified with Agencourt AMPure XP beads and submitted to the CIBIR Genomics Core Facility (La Rioja, Spain) for quality control (Fragment Analyzer, Agilent) and quantification (Qubit HS DNA Kit, ThermoFisher). Barcodes (Nextera XT, Illumina) were added before sequencing on a MiSeq System (Illumina, PE300 model).

FASTQ files provided from the sequencing facility were assessed for quality using FastQC (v0.12.1), trimmed with Cutadapt (v5.0), and processed using DADA2 (v1.30.0) to generate Amplicon Sequence Variants (ASVs). Taxonomic classification was performed using QIIME2 (v2025.4) with the SILVA v138.2 database as reference. Non-bacterial ASVs were filtered out to minimize bacterial diversity overestimation. Ecological analyses were conducted in R 4.3.1 (v2023.06.16). The Phyloseq package was used for data integration and handling. Alpha and beta diversity metrics were computed with Vegan, and visualized with ggplot2.

### Gnotobiotic plant germination and growth

Disinfected seeds were placed on 1% agar plates with 1 ml sterile distilled water and incubated in the dark at 4°C 48 hours (*Arabidopsis*) or overnight (Tae_SP2) for stratification. All plates were then incubated at 24°C in the dark for 48 hours to allow germination. Seedlings were individually transferred to sterilized glass tubes containing 20 ml Murashige and Skoog Basal salt mixture (Sigma:M5519) and a filter paper. Tubes were sealed with cotton, covered, and placed in a growth chamber (16/8-hour light/dark, 24°C, 60% humidity). After 7 days, the shoots and roots of three individual seedlings were independently excised under sterile conditions for DNA extraction and amplicon sequencing.

### GUS labelling of *Pantoea agglomerans*

Plasmid pGUS-3 [38] was conjugated into *P. agglomerans* C-88, isolated from surface disinfected *Triticum spelta* seeds (Tpe_SP4), via biparental mating with *Escherichia coli* S17–1 (λpir). The conjugation mixture was plated on TSA with kanamycin (50 μg/ml) and incubated overnight at 28°C. Transconjugants were transferred to TSA plates containing the chromogenic substrate X-gluc (1 μl/ml, Biogen). Hydrolysis of X-gluc by β-glucuronidase encoded by pGUS-3 resulted in blue colonies after overnight incubation at 28°C. Presence of the plasmid was confirmed by PCR amplification using GUS_F and GUS_R primers (Table S4), yielding strain *P. agglomerans* C88-GUS.

### Plant growth conditions, C88-GUS inoculation and detection

*P. agglomerans* C88-GUS overnight cultures grown on TSA were suspended in PBS at an OD_600nm_ of 0.6 (~4 × 10^8^ Colony Forming Units [CFUs]/ml). G0 surface-disinfected seeds of wheat, *Lolium*, and *Arabidopsis* were germinated as described. Seedlings were transferred to pots with a 50% soil-vermiculite mixture and grown in a greenhouse (16/8-hour light/dark photoperiod, 18–30°C, 60% relative humidity) until flowering.

For bacterial incorporation into seeds, *Arabidopsis* flowers were individually sprayed with 50 μl of the C88-GUS suspension, followed by removal of the floral bud. For *Lolium* and wheat, spikes were immersed in 40 ml of the same suspension for 1 min. To assess bacteria transmission across generations, G0 seeds were imbibed in a C88-GUS suspension for 24 hours, sown into pots, and grown in the greenhouse until flowering. Seeds from the subsequent G1 generation were harvested and stored at 4°C until further analysis. All control plants were treated with PBS without bacteria. C88-GUS was qualitatively (GUS staining) and quantitative (qPCR) detected in 7-day-old seedlings.

## Results

### *Pantoea* is the only core taxon present in wheat seed species

**Figure 1:**
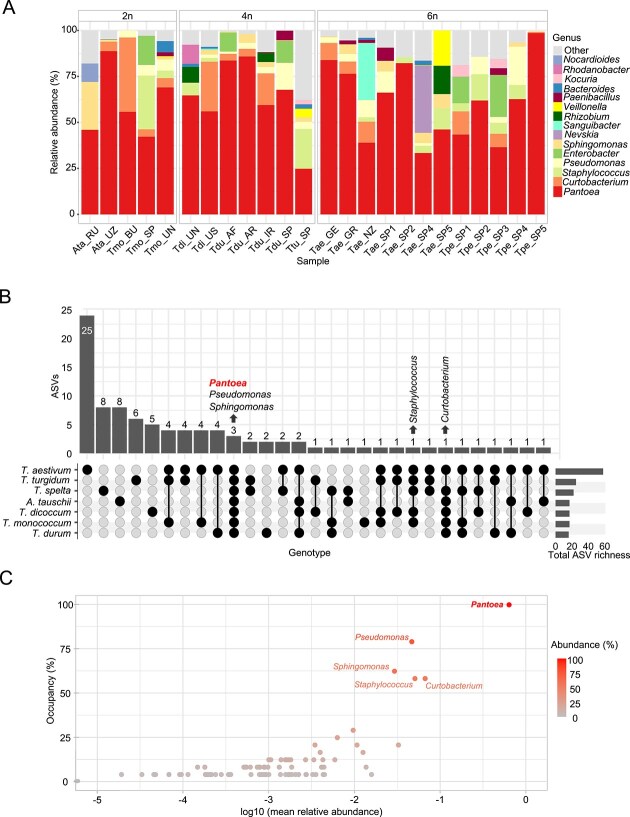
Endophytic bacterial diversity of worldwide wheat seeds. (A) Relative abundance of the 15 most dominant bacterial genera in the endophytic seed microbiota of diploid (2n) wheat species *Aegilops tauschii* (Ata) and *Triticum monococcum* (Tmo); tetraploid (4n) *Triticum turgidum* (Ttu), *Triticum dicoccum* (Tdi), and *Triticum durum* (Tdu); and hexaploid (6n) *Triticum aestivum* (Tae) and *Triticum spelta* (Tpe) (see Tables S1 & S2). Each bar represents a pooled sample of 12 seeds. (B) UpSet plot showing shared and unique bacterial ASVs among the seven wheat species. ASVs were included if detected in at least one sample per wheat species. Taxonomic assignments at the genus level are shown for ASVs shared by at least five wheat species. (C) Abundance–occupancy distribution of bacterial ASVs. Each point represents an ASV, with occupancy (proportion of samples in which it was detected) on the y-axis and mean relative abundance (log₁₀ scale) on the x-axis. Intensity denotes relative abundance. ASVs detected in >50% of samples were taxonomically assigned at the genus level. The core *Pantoea* ASV is highlighted in bold.

To assess bacterial endophytic communities, first we set a reliable protocol to ensure total removal of bacterial seed external load (Fig. S1) by sonication followed by a surface-disinfection published method [34]. Next, to identify core seed endophytes, we analyzed 24 globally sourced wheat samples (Tables S1 and S2) and found that seed endophytic bacterial communities are relatively conserved at genus level, with *Pantoea* as the dominant group (Fig. 1A).

Besides *Pantoea* (61% average abundance), the most common genera were *Curtobacterium* (6%), *Staphylococcus* (5%), and *Pseudomonas* (3%). We grouped samples by ploidy level and domestication status for ANCOM-BC2 analysis. Only *Pseudomonas*, *Paenibacillus*, and *Staphylococcus* showed significantly higher abundance in commercial compared to ancestral seed samples.

Seed endophytic communities showed low α-diversity and no significant differences in composition across domestication groups, species, genome content, or geographic origin.(Wilcoxon rank-sum test, Fig. S2). The PCoA plot revealed no clear clustering or patterns in relation to these sample characteristics (Fig. S3). Full and individual PERMANOVA analysis did not show statistical significance, further supporting the conservation of seed endophytic assemblages across wheat varieties worldwide.

To identify commonalities, the 92 seed bacterial ASVs present in each of the seven wheat species were used to generate an Upset plot (Fig. 1B). Most ASVs were species-specific, with *T. aestivum* showing the highest number of unique ASVs (25). Taxonomic assignment at the genus level revealed that *Pantoea*, *Pseudomonas*, and *Sphingomonas* were consistently present across all species, whereas *Curtobacterium* and *Staphylococcus* appeared in most.

To identify bacterial core taxa shared across seeds from different wheat species, we used a distribution illustrating the relationship between average abundance and occupancy across taxa (Fig. 1C). Most taxa exhibited low occupancy, suggesting sporadic presence and likely representing transient or rare community members. In contrast, several taxa showed high occupancy and substantial relative abundance including *Pseudomonas, Sphingomonas, Curtobacterium,* and *Staphylococcus*. *Pantoea* was the only taxon consistently detected in samples (occupancy = 100%), highlighting it as a highly prevalent, abundant, and core taxon across species.

### *Pantoea* is the most abundant genus in gnotobiotically germinated wheat seedlings

**Figure 2:**
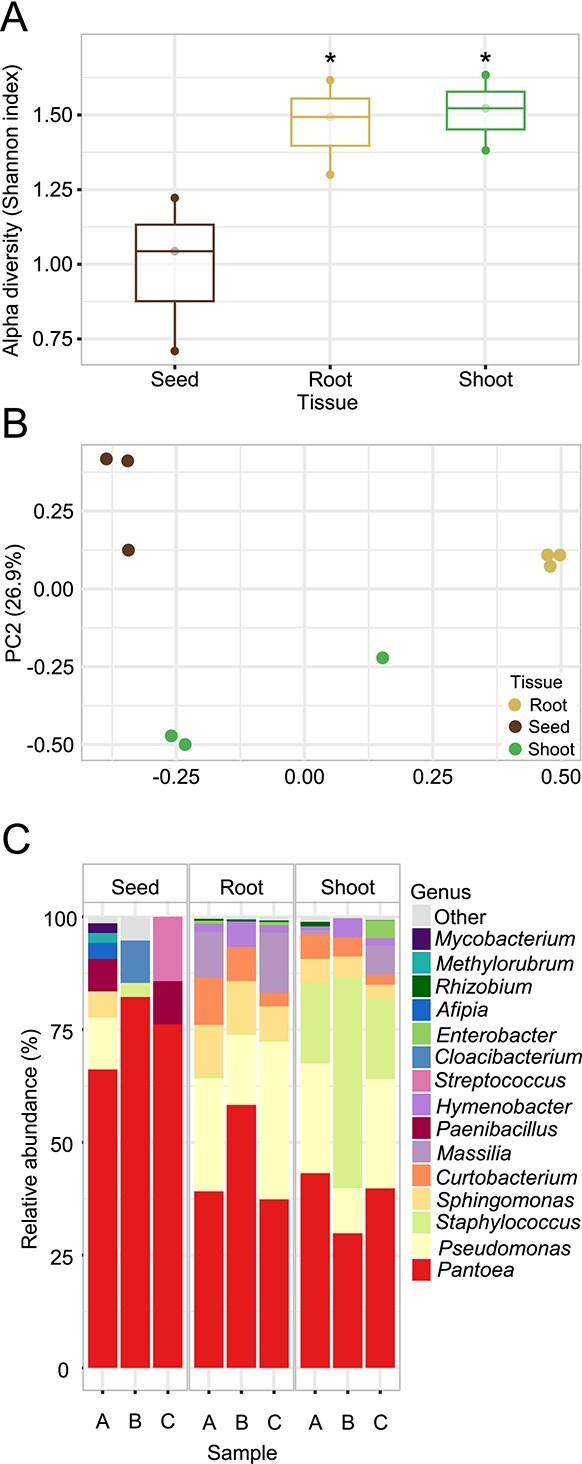
Endophytic bacterial diversity of wheat seedlings cultured under gnotobiotic conditions. (A) Shannon index of Tae_SP2 surface disinfected seeds and gnotobiotically germinated shoot and roots. Each boxplot show the distribution of Shannon diversity index, with the median, interquartile range (IQR), and potential outliers. ^*^P < .05 compared to seeds (Tukey’s post-hoc test). (B) PCoA corresponding to the Bray–Curtis dissimilarity index (β-diversity) of the bacterial communities present in the different plant tissues. Each dot corresponds to an individual technical replicate. The x- and y-axes represent the first and second components of the PCoA plot, respectively. PERMANOVA: *R*^2^ = 0.65, *P* = .002. (C) Relative abundance of the 15 most prevalent bacterial genera present in wheat seeds and gnotobiotically germinated seedlings. Three independent sample replicates are shown (A, B, C; n = 3). Each seed replicate corresponds to a pool of 12 surfaced-disinfected seeds from the same wheat variety sample (Tae_SP2, Table S1), whereas shoots and roots samples represent three independent biological replicates from three different plants.

We determined seed-to-seedling transmission and core community dynamics after germination under gnotobiotic conditions using a hydroponic cultivation system. Microbial communities from roots and shoots of Tae_SP seedlings obtained by 16S rRNA gene sequencing were compared with those from non-germinated seeds. The diversity of the detectable bacterial community in three seedling samples was significantly higher than that observed in seeds (Fig. 2A). Furthermore, seed samples clustered separately from the three independent post-germination communities (Fig. 2B). However, community composition did not differ significantly between these tissues. Most genera that are present in the seeds at low percentages proliferated upon germination in roots or/and shoots, like *Curtobacterium* (Fig. 2C). *Pantoea* was the only prevalent ASV whose average relative abundance in seeds (75% in these 3 sample sets) decreased in germinated roots and shoots (45 and 38%, respectively). Nevertheless, *Pantoea* remains the most relative abundant bacterial member in both wheat roots and shoots 7 days after germination.

### All wheat tissues grown under field conditions contain *Pantoea*

**Figure 3:**
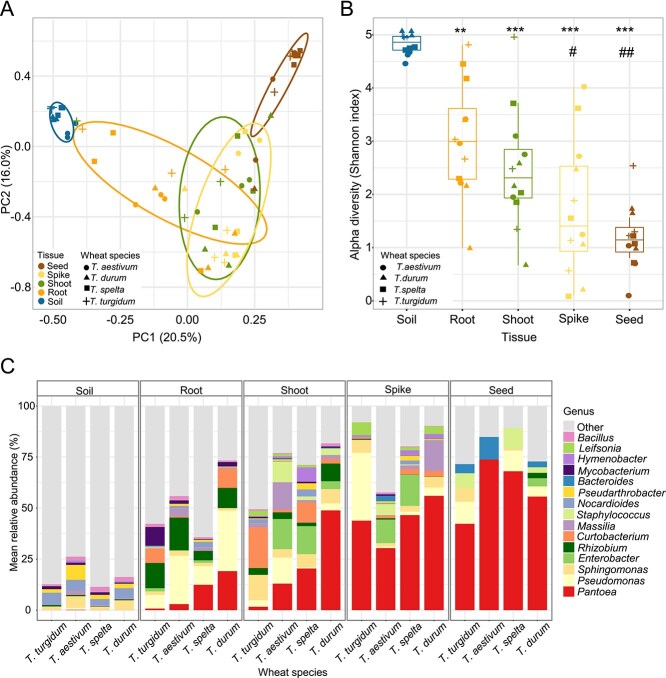
Endophytic bacterial diversity of wheat tissues and species grown under field conditions. (A) Shannon index of different wheat plant tissues and the surrounding soil. Each dot corresponds to an individual biological replicate. Seeds (pools of 12) and plant tissues were collected per parent plant. Statistical significance (Tukey’s post-hoc test): ^**^P < .001; ^***^P < .0001 versus soil; ^#^  *P* < .01; ^##^  *P* < .001 versus root. Each boxplot show the distribution of Shannon diversity index, with the median, interquartile range (IQR), and potential outliers. (B) β-diversity (PCoA) of the bacterial communities associated with different plant tissues (PERMANOVA *R*^2^ =0.33, *P* = .001) and wheat species (*R*^2^ = 0.05, *P* = .4) as indicated in the legend. Each dot corresponds to an individual biological replicate. Ellipses represent the clustering and relative homogeneity of bacterial communities within tissues. (C) Relative abundance of the 15 most dominant bacterial genera across soil and plant tissues of *Triticum turgidum, Triticum aestivum, Triticum spelta*, and *Triticum durum*. Data are the average of three individual biological replicate or three pools of 12 seeds (n = 3) collected in different plot sites from the same location.

We compared the communities inhabiting seeds and plant tissues and rhizosphere of four wheat species (Table S3) grown in the field to assess core endophytic bacterial dynamics under natural conditions. Alpha-diversity analysis revealed significant differences between soil and all plant-derived communities, as well as between roots and both seed and spikes (Fig. 3A). The seed community exhibited the lowest diversity, which increased progressively in the order of seed < spike < shoot < root < soil. β-diversity (Fig. 3B) revealed that bacterial communities significantly differed across plant tissues (*R*^2^ = 0.33, *P* = .001), whereas plant genotype had no effect (*R*^2^ = 0.05, *P* = .497). The interaction between both factors verified the influence of compartment component (*R*^2^ = 0.33, *P* = .001) over genotype factor (*R*^2^ = 0.05, *P* = .048; residual *R*^2^ = 0.62). Taxonomic profiles mirrored these trends, with diversity decreasing from soil to seeds (Fig. 3C).

**Figure 4:**
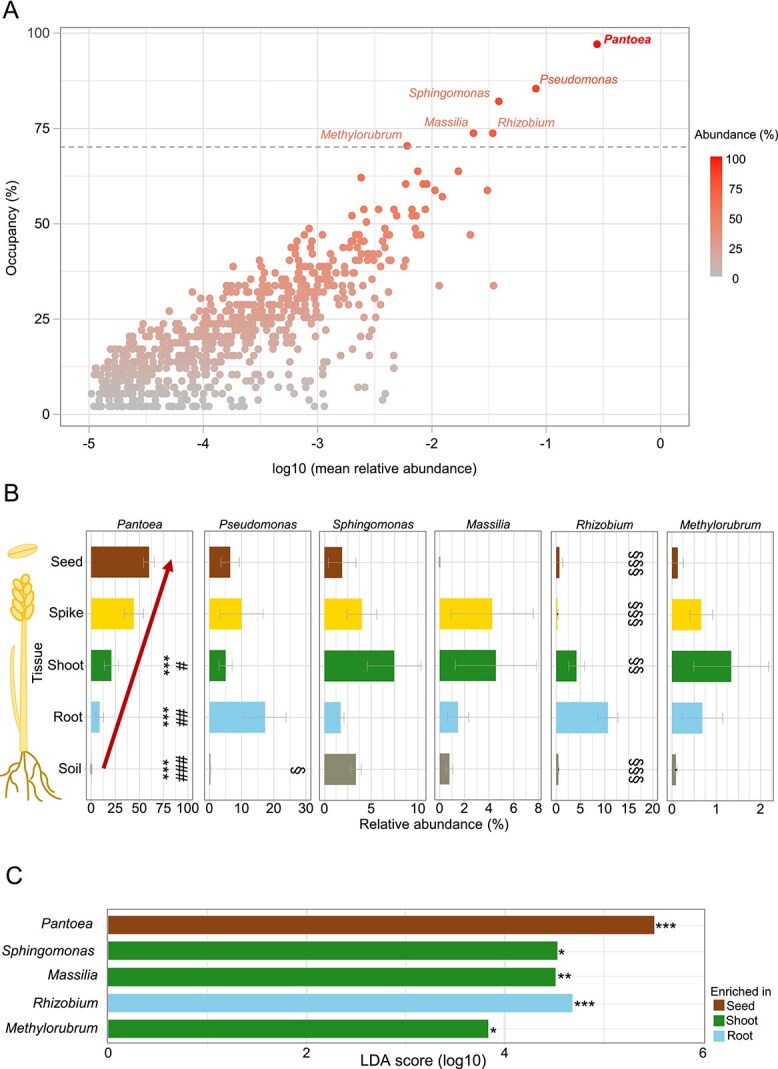
Wheat endophytic bacteria dynamics during plant field development. (A) Abundance–occupancy distribution of bacterial ASVs detected across all field samples (see Fig. 1C for details). ASVs present in >70% of the samples were assigned to the genus level. (B) Average relative abundance of ASVs with occupancy >0.7 (panel A) in soil and wheat tissues. Error bars represent SEM of three biological replicates. Statistical significance (Tukey’s post-hoc test): ^***^P < .001 versus seed; ^#^  *P* < .01; ^##^  *P* < .001 and ^###^  *P* < .001 versus spike; and ^**§**^  *P* < .01; ^**§§**^  *P* < .001 and ^**§§§**^  *P* < .001 versus root. (C) LDA effect size of taxa with occupancy >0.7 (panel A), showing those differentially enriched in seeds, spikes, shoots, or roots. Only taxa with LDA score > 2 and adjusted *P* < .05 are shown; bars are colored by enriched compartment. Statistical significance: ^*^P < .01; ^**^P < .0001; ^***^P < 1 × 10^−7^.

Vertical transmission is likely a common feature among seed-ubiquitous bacteria that also colonize other plant tissues. Among these, *Pantoea* also exhibited the highest occupancy (96%) and relative abundance across compartments (Fig. 4A, in bold). Other highly prevalent taxa (occupancy >70%) with substantial relative abundance included *Pseudomonas, Sphingomonas, Rhizobium, Massilia,* and *Methylorubrum*.

### *Pantoea* abundance shows a distinct upward pattern from root to seed wheat tissues

To identify potential signs of vertical transmission, we studied the prevalent taxa among the four wheat species and tissues. Population dynamics of *Pantoea,* which was not detected in most soil samples, exhibited the lowest relative abundance in roots and increased progressively in the order of root < shoot < spike < seed in all analysed wheat species (Fig. 3C). This upward gradient was particularly pronounced in *T. aestivum* and *T. spelta*, where relative abundances of *Pantoea* increased sequentially from root to seed, ranging from 2%–11%–25%–61% to 8%–14%–32%–46%, respectively. We compared enrichment patterns of the six taxa with occupancy >0.7 across plant tissues and soil (Fig. 4A). Mean prevalence across wheat species in roots, shoots, spikes, seeds, and the soil confirmed that *Pantoea* displayed a clear vertical enrichment with significantly higher relative abundance in seeds and spikes compared to other compartments and soil (Fig. 4B). In contrast, *Pseudomonas* and *Rhizobium* were most abundant in roots. LEfSE analysis further revealed that *Pantoea* showed a significant enrichment in seeds, whereas *Sphingomonas, Massilia,* and *Methylorubrum* in shoots (Fig. 4C). This analysis identified *Pantoea* as the most enriched genus in seeds among all genera detected in wheat field samples (linear discriminant analysis [LDA] score 5.5, *P* = 2 × 10^−8^). Therefore, *Pantoea* is the only prevalent taxon exhibiting a consistent vertical enrichment gradient (Fig. 4B, red arrow).

### Vertical transmission of *Pantoea* to the offspring plant generation through the seeds

**Figure 5:**
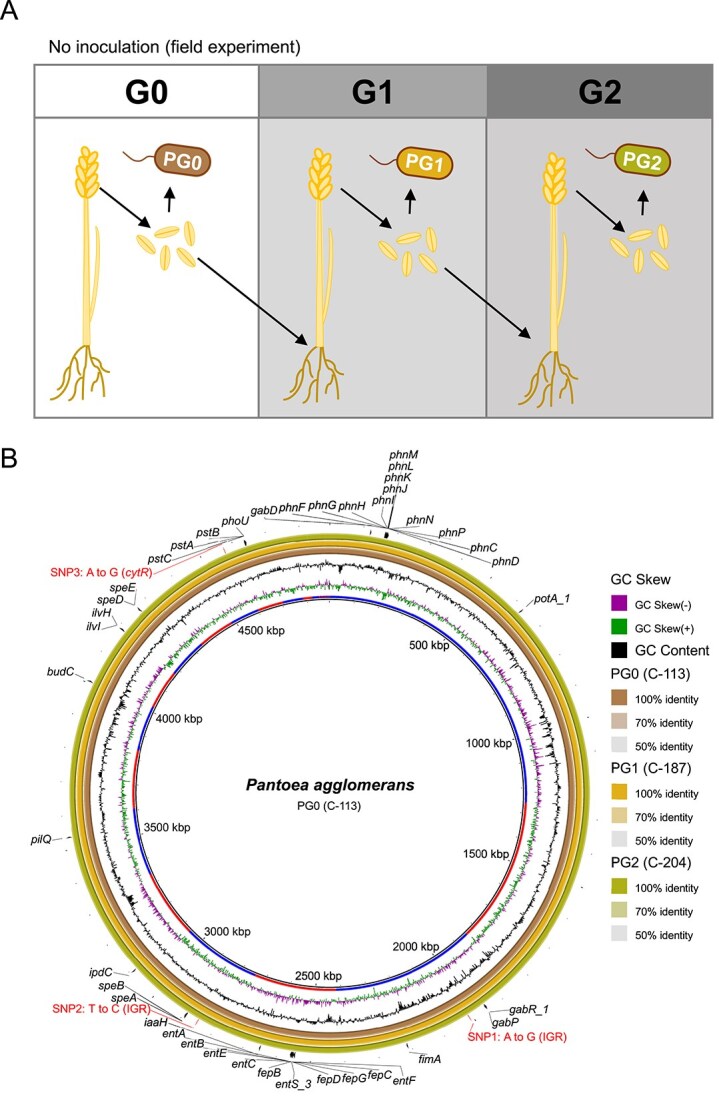
Genomic comparison of *Pantoea agglomerans* seed isolates across three wheat generations*.* (A) Schematic representation of three successive wheat generations cultivated in the field (G0, G1, and G2). Seed-derived *P. agglomerans* isolates (PG0, PG1, PG2) from each generation were subjected to whole-genome sequencing. (B) Circular BLAST ring image generator plots illustrating high genomic similarity among the three isolates. From the innermost to the outermost ring: (i) contig boundaries (alternating segments) indicating assembly structure, (ii) GC skew, and (iii) GC content illustrating compositional variation, (iv–vi) BLASTn comparisons with PG0 (*P. agglomerans* C-113), PG1 (*P. agglomerans* C-187), and PG2 (*P. agglomerans* C-204), and (vii) SNPs found between PG0 and PG2 genomes (Table S5) and gene annotations identified in PG0 genome potentially involved in plant growth promotion (Table S6).

**Figure 6:**
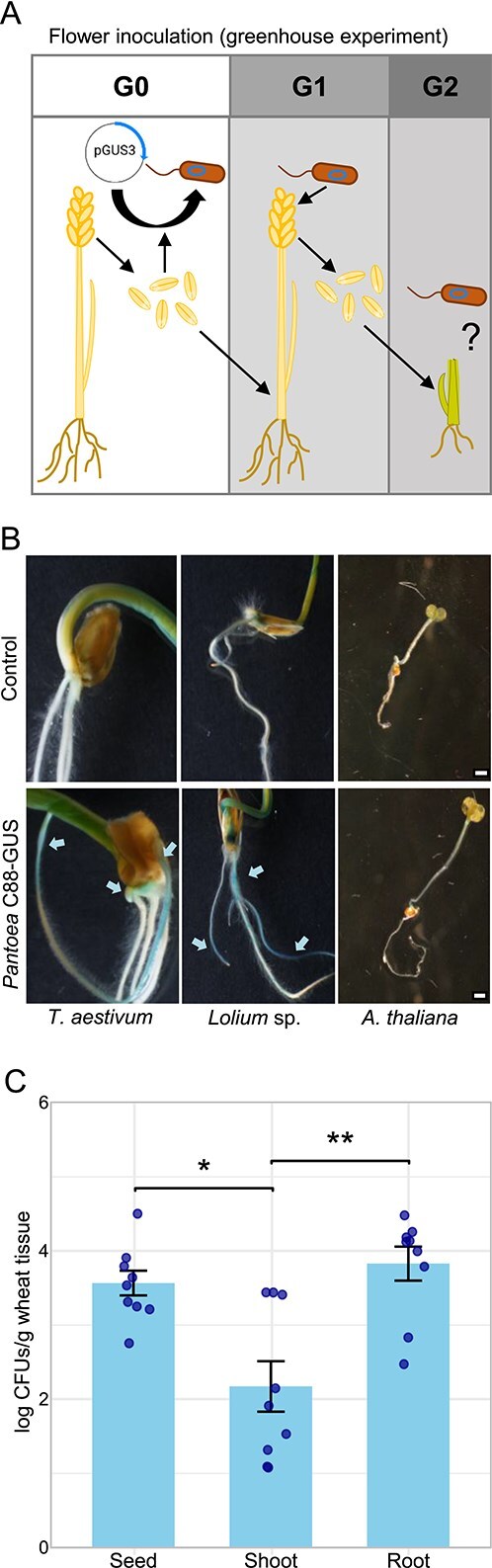
*Pantoea* seed incorporation and seed-to-seedling transmission in *Triticum aestivum* and *L. multiflorum*. (A) Schematic of the first set of greenhouse experiments: Flowers were inoculated with a GUS-labelled *Pantoea agglomerans* seed isolate, and seeds of the G2 progeny were germinated and stained to detect bacterial colonization. (B) Detection of *Pantoea* colonization in seedlings by X-gluc histochemical staining. Arrows indicate the presence of *Pantoea* C88-GUS in *T. aestivum* and *L. multiflorum* (Poaceae). (C) Quantification of *Pantoea* populations in wheat tissues by qPCR. Error bars represent the standard error of the mean from three independent experiments, each with three biological replicates. Scale bar 5 mm. Statistical significance (Tukey’s post-hoc test): ^*^P < .01 ^**^P < .001.

We hypothesize that *Pantoea* is vertically transmitted via seeds to subsequent generations, supported by its consistent presence and abundance in seeds and its enrichment across plant tissues. To test this, we cultivated *T. aestivum* over three generations in the field (Fig. 5A). Seed endophytic microbiota of each generation (G0 to G2) was cultured, and *Pantoea* isolates were identified, with most classified as *P. agglomerans*. WGS of three *P. agglomerans* isolates belonging to the three generations (PG0 to PG2) revealed strong genomic similarity (Fig. 5B), with only a single SNP located in a CDS (Table S5) differentiating PG0 (C-113) and PG2 (C-204). Genomic surveillance of these strains revealed the presence of genes potentially involved in plant growth promotion (Fig. 5B, Table S6), consistent with previously described beneficial *P. agglomerans* strains. Isolation of nearly identical *Pantoea* strains across generations supports the hypothesis of vertical transmission through wheat seeds. To experimentally confirm this theory, we tracked a GUS-labelled *Pantoea* wheat seed isolate (C-88) across plant generations. In a first set of greenhouse experiments (Fig. 6A), wheat spikes were inoculated with *Pantoea* C88*-*GUS, and the resulting seeds were harvested. Upon germination, 7-day-old wheat seedlings grown from these seeds exhibited clear blue GUS staining, in contrast to the roots of uninoculated control plants, which showed no staining (Fig. 6B). *Pantoea* quantification in these G1 germinated seedlings was performed through qPCR by interpolating Ct values in a standard curve (Fig. 6C). Prior to DNA extraction, G1 seedlings were split into shoot, root, and the remaining seed. Matching the colorimetric results, *Pantoea* C88*-*GUS preferably colonized G1 wheat roots. To investigate if *Pantoea* vertical transmission ability was conserved in a plant phylogenetic framework, we performed the same experiment with seeds from the forage grass *L. multiflorum* (*Poaceae*) and the model plant *A. thaliana* (Brassicaceae). GUS staining confirmed *Pantoea* incorporation and transmission in *Lolium* but not in *Arabidopsis* (Fig. 6B).

**Figure 7:**
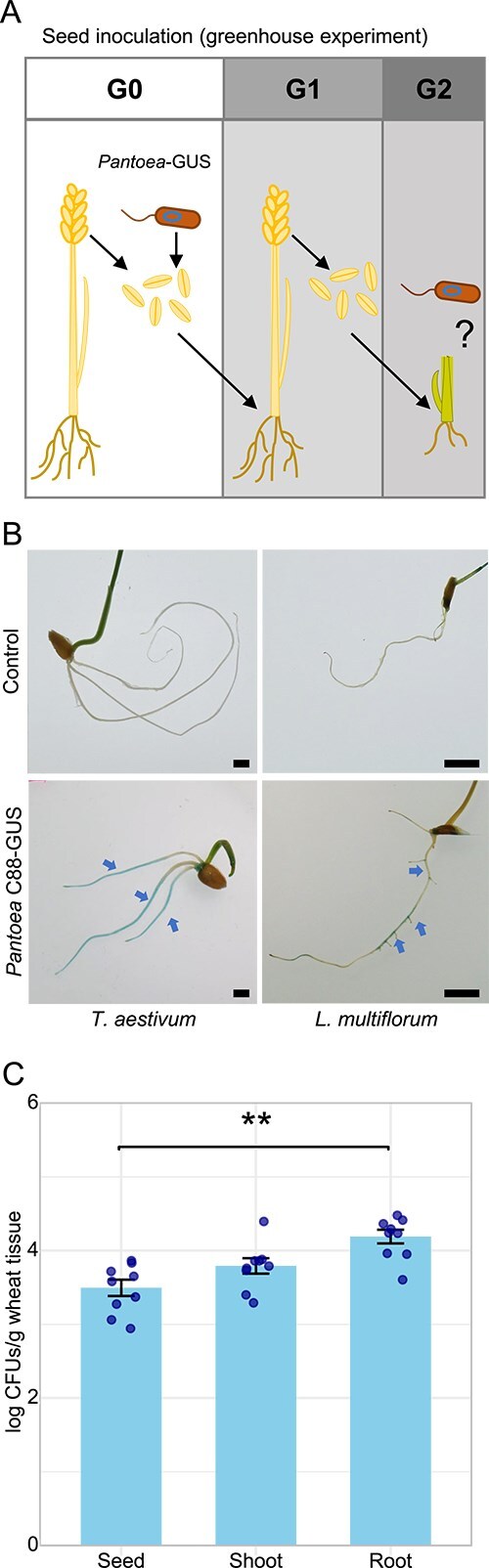
Experimental validation of *Pantoea* vertical transmission from seeds to seedlings of the G2 progeny. (A) Schematic of the second set of experiments: Seeds were imbibed with the GUS-labelled *Pantoea agglomerans* isolate and grown until flowering; seeds from the next generation were germinated and stained to detect bacterial colonization. (B) Detection of *Pantoea* colonization in seedlings by X-gluc histochemical staining. Arrows indicate the presence of *Pantoea* C88-GUS. (C) Quantification of *Pantoea* populations in wheat tissues by qPCR. Error bars represent the standard error of the mean from three independent experiments, each with three biological replicates. Scale bar 5 mm. Statistical significance (Tukey’s post-hoc test): ^**^P < .001.

Both qualitative and quantitative methods support that *Pantoea* can be incorporated to the seeds of wheat and transmitted to the germinating seedlings. Finally, to confirm bacterial vertical inheritance from seeds to seedlings of the G2 progeny we set up a new set of experiments (Fig. 7A). *Lolium multiflorum* and *T. aestivum* G0 seeds were imbibed with the labelled endophyte and germinated seedlings were transferred to plant pots until flowering. After harvest, the next generation of seeds (G1) were germinated again, and G2 seedlings were stained with X-gluc to verify the presence of the endophyte as described before (Fig. 7B). The results obtained by GUS histochemical analysis and qPCR methods matched and show that C88-GUS accumulated significantly in the root seedlings, reaching 1.25 × 10^4^ CFUs per gram of tissue (Fig. 7C). Collectively, these results confirm that *Pantoea* C88-GUS can be vertically inherited to the offspring generation through the seeds in both *Poaceae*.

## Discussion

Seed microbial reservoir is involved in bacterial transmission, as inferred from shared species in seeds and seedlings [28–31, 39]*.* However, the presence of common species does not necessarily implicate vertical transmission, as considerable heterogeneity exists within taxa from the same species. Experimental confirmation by inoculation is needed to verify steady vertical transmission of plant-associated bacteria. This study demonstrates vertical transmission events of an endophyte through plant generations providing insights into the acquisition and community dynamics of the seed microbiome. Our findings uniquely contribute to advancing this field because they rigorously prove for the first-time evidence of endophyte transmission across three plant generations.

We hypothesized that core endophytic seed bacteria widespread among a worldwide-cultivated cereal like wheat will be ideal candidates for confirming vertical transmission. Most studies aiming to widely characterize the seed-associated microbiota do not perform a profound surface-disinfection of the seeds [40]. The role and diversity of bacteria inhabiting seed endosphere is yet under-explored, mainly due to technical difficulties and the absence of standardized methodologies [25, 41]. Therefore, we firstly aimed to establish a robust surface disinfection protocol to eliminate the abundant seed epiphytes. Our experiments provide evidence that sonication and a previously published method [34] protocol led to reliable seed surface-disinfection, removing epiphytic bacteria. This protocol for analyzing seed endophytes can be applied to other plant species.

As corroborated by previous literature, seeds harbor low-diverse endophytic bacterial communities, mostly dominated by Gamma-Proteobacteria and Firmicutes [14, 26, 42]. α-and β-diversity analyses suggest that harvest location, wheat species, genome content, or domestication level do not majorly influence bacterial diversity, supporting the absence of a rational taxonomical structure as suggested before [27]*.* Previous studies also showed no significant differences between commercial and ancestral wheat seed-derived communities [14], whereas others reported higher diversity among cultivated cereals [43]. However, the differences in plant varieties, seed disinfection, and sequencing methods hamper reliable comparisons. In agreement with our results, a recent meta-analysis of 63 seed microbiota studies reported that *Pantoea* was one of the most abundant and prevalent seed-borne genera, being present in 27 different plant species [44]. This suggests that these members of the endophytic communities may be essential for development and adaptation of different plant species.

*Pantoea*, belonging to *Erwiniaceae*, is a diverse facultatively anaerobic genus of yellow-pigmented bacteria. *Pantoea* comprises over 20 recognized species that show a remarkable ecological adaptability, being frequently isolated from diverse environments, especially in association with plants [45, 46]. Whereas some species, *lik*e *Pantoea stewartii,* show pathogenic traits [47]; *other like P. agglomerans* possess plant growth-promoting and biocontrol abilities [45]. This species was the most frequently isolated in our wheat seed samples.

Microbial succession during germination differs between plant species [25, 26, 40, 48]. Our gnotobiotic system confirms that not all wheat seed endophytic taxa are necessarily transmitted to seedlings [26, 42]. In the absence of bacteria from the rhizosphere, the dominance of *Pantoea* is maintained in plant roots and shoots upon germination. In contrast, we noticed an increase in bacterial taxonomic diversity in wheat roots and shoots grown under field conditions, matching the previously reported replacement of this dominant pioneer seed endophytic taxa by soil-derived microorganisms [27]. *Pantoea* persisted across wheat tissues from all wheat species that we scrutinized, and its relative abundance gradually increases from roots to seeds. Our results also indicated that relative abundances of other common bacterial genera did not show a *Pantoea*-like consistent gradient across wheat tissues. These results suggest that wheat microbial community assembly is tissue-specific, as described before in other plant species and tissues [49–51]. Nevertheless, this intriguing accumulation pattern may not be necessary for vertical transmission and other endophytes capable of colonizing some wheat seeds may be also transferred over generations. Environmental drivers and host pressures shaping wheat microbiota warrant further study [52].

*Pantoea* unique vertical enrichment through wheat plant tissues and the isolation of *P. agglomerans* strains with almost identical genomes across three wheat generations supports the hypothesis of transfer of these endophytic bacteria through the seeds. To experimentally confirm intergenerational transmission of endophytes, we tracked the colonization ability of the wheat seed isolate *P. agglomerans* C-88 across plant generations. Our results confirm that bacterial endophytes can be vertically transferred via seeds to the progeny through plant generations. GUS-tagged isolates were detected in *T. aestivum* and *L. multiflorum* (*Poaceae*)*,* but not in *Arabidopsis thaliana* (*Brassicaceae*), suggesting host-specificity in vertical transmission. This hypothesis is consistent with previous studies showing that seed-associated microbiota exhibits host specificity, which may be driven by co-evolutionary dynamics, selective pressures imposed by the host plant morphology, chemistry and immune system, or by distinct bacterial transmission pathways [27, 32]. *Pantoea* was detected in non-disinfected seeds, roots, and shoots of *Arabidopsis* grown in closed jars [27]. Additionally, *P. agglomerans* was also one of the three bacterial OTUs detected in all radishes (*Brassicaceae)* unsterilized seed samples across sequencing of three successive plant generations [29]*.* These findings support the occurrence of vertical transmission of *P. agglomerans* in certain Brassicaceae species. Consequently, further testing is needed to assess the vertical transmission potential of diverse *P. agglomerans* strains in additional *Brassicaceae* hosts [6, 22].

*Pantoea* was not detected in most wheat rhizospheric soil samples, further supporting that it is vertically transmitted through wheat seeds. This finding aligns with the theory that vertically-transmitted endophytes may form stable, co-evolved relationships with their host plants [6]. *Pantoea* ability to be transmitted to the progeny has also been well-documented in insects [53]. Some Hemiptera establish obligate symbiotic associations with a wide diversity of *Pantoea,* which are vertically inherited from adult females to nymphs. These symbiosis-like associations may be shaped by selective pressures that favor non-pathogenic strains [54], together with the benefits gained by both the bacteria and the host. The microbial counterpart ensures its dispersion, survival, and niche displacement of microbial competitors, whereas the host benefits by providing their progeny with beneficial symbionts [21]*.* Plant microbiome functions like germination are essential for some hosts [55]. Additionally, cosmopolitan plant-associated bacteria, including *Pantoea, Stenotrophomonas, Bacillus,* and *Pseudomonas*, positively impact germination [56]. In particular, treatment of seeds with *P. agglomerans* PS1 significantly increased wheat seed germination (up to 25%), plant development, and grain production [57]. Similar to the PS1 strain, our *P. agglomerans* wheat seed isolates also harbor genes associated with phosphate solubilization and other plant growth promotion activities like auxin and siderophore biosynthesis. Furthermore, we identified several genes potentially involved in plant colonization, including those encoding proteins required for the synthesis of pili. Genes associated with the biosynthesis of volatile organic compounds, polyamines, and γ-aminobutyric acid were also present. These compounds have been implicated in enhancing root development, promoting systemic disease resistance, and contributing to pathogen inhibition [58]. Altogether, these findings suggest that *Pantoea* may establish a beneficial symbiotic relationship favouring both germination and plant development.

The fact that *Pantoea* was the only genus identified in all our analyzed wheat seed samples agrees with the taxonomical restriction of microorganisms that seem to consistently pass on to progeny plants [29, 59]*.* This further suggests that the ability to be transmitted from seed to seed across plant generations is not a widespread trait among bacteria and that there must be strict filtering processes governing these mechanisms. Similarly, some microorganisms are directly transferred from mother to baby during birth, although few persist from birth to adulthood in the offspring [60]. Investigation into these mechanisms deserves further studies. It is tempting to speculate that plant defense against seed-derived pathogens, along with the unique morphological and chemical characteristics of seeds as a bacterial niche, may play a role in these filtering processes.

To our knowledge, only the leaf-nodulating nitrogen-fixing *Burkholderia* symbionts were described as obligate in plants [61, 62]. However, *Burkholderia*-free host plants survived in a sterile in vitro environment [23], suggesting that even for obligate symbionts vertical transmission may not be the only route for plant survival. The ability of surface sterilized wheat embryos to germinate leading to axenic seedlings [7], suggests the absence of obligate endophytes also in these cereal plants, at least under laboratory conditions.

Our findings suggest that transmission from parent to progeny is a common strategy among *Pantoea* endophytes, but the genetic and ecological factors driving host-specificity require further research [6]. The conservation and dynamics of this microbial genus suggests a potential evolutionary symbiosis-like relationship maintained between *Pantoea* and wheat throughout its domestication process. This host-microbiota co-evolution aligns with the plant holobiont theory, which posits that plant fitness is closely linked to its associated microbiota and that plants, in turn, can actively shape their microbial communities to dynamically adapt to environmental changes [63].

Our results confirm that vertical transfer of bacterial endophytes occurs, but whether obligate and strictly vertically transferred symbioses with bacteria is a widespread phenomenon remains obscure. Future studies exploring the co-evolution of plants and their microbial partners in early developmental stages could provide valuable insight into the long-term stability and functional significance of these symbiotic interactions.

## Supplementary Material

Supplementary_materials_wraf192

## Data Availability

Raw reads from WGS are available in the NCBI GenBank repository under the *accession* number PRJNA1201053. 16S rRNA gene amplicon sequencing raw data generated, along with corresponding sample metadata, in this study have been included as a supplement to this publication and have been deposited in the NCBI Sequence Read Archive under the BioProject accession number PRJNA1282304. The scripts used to analyzed the data and generate the figures are available on GitHub: https://github.com/MartaRobledoLab/Sanz-Puente_et_al_2025.

## References

[1] Araujo G, Montoya JM, Thomas T. et al. A mechanistic framework for complex microbe-host symbioses. *Trends Microbiol* 2024;33:96–111. 10.1016/j.tim.2024.08.002 39242229

[2] Liu S, Zhang Z, Ma L. A review focusing on microbial vertical transmission during sow pregnancy. *Vet Sci* 2023;10:123. 10.3390/vetsci10020123 36851427 PMC9967962

[3] Trivedi P, Leach JE, Tringe SG. et al. Plant–microbiome interactions: from community assembly to plant health. *Nat Rev Microbiol* 2020;18:607–21. 10.1038/s41579-020-0412-1 32788714

[4] Hassani MA, Durán P, Hacquard S. Microbial interactions within the plant holobiont. *Microbiome* 2018;6:58. 10.1186/s40168-018-0445-0 29587885 PMC5870681

[5] Durán P, Thiergart T, Garrido-Oter R. et al. Microbial Interkingdom interactions in roots promote *Arabidopsis* survival. *Cell* 2018;175:973–983.e14. 10.1016/j.cell.2018.10.020 30388454 PMC6218654

[6] Campisano A, Berg G, van Overbeek LS. et al. The hidden world within plants: ecological and evolutionary considerations for defining functioning of microbial endophytes. *Microbiol Mol Biol Rev* 2015;79:293–320. 10.1128/MMBR.00050-14 26136581 PMC4488371

[7] Robinson RJ, Fraaije BA, Clark IM. et al. Wheat seed embryo excision enables the creation of axenic seedlings and Koch’s postulates testing of putative bacterial endophytes. *Sci Rep* 2016;6:25581. 10.1038/srep25581 27151146 PMC4858700

[8] Robledo M, Jiménez-Zurdo JI, Velázquez E. et al. *Rhizobium* cellulase CelC2 is essential for primary. *Proc Natl Acad Sci USA* 2008;105:7064–9. 10.1073/pnas.0802547105 18458328 PMC2383954

[9] Gutiérrez-Zamora M, Martinez-Romero E. Natural endophytic association between *Rhizobium etli* and maize (*Zea mays* L.). *J Biotechnol* 2001;91:117–26. 11566384 10.1016/s0168-1656(01)00332-7

[10] García-Fraile P, Carro L, Robledo M. et al. *Rhizobium* promotes non-legumes growth and quality in several production steps: towards a biofertilization of edible raw vegetables healthy for humans. *PLoS One* 2012;7:e38122. 10.1371/journal.pone.0038122 22675441 PMC3364997

[11] Nelson EB . The seed microbiome: origins, interactions, and impacts. *Plant Soil* 2018;422:7–34. 10.1007/s11104-017-3289-7

[12] Shade A, Jacques MA, Barret M. Ecological patterns of seed microbiome diversity, transmission, and assembly. *Curr Opin Microbiol* 2017;37:15–22. 10.1016/j.mib.2017.03.010 28437661

[13] Cardinale M, Schnell S. Is the plant microbiome transmitted from pollen to seeds? *Front Microbiol* 2024;15:1343795. 10.3389/fmicb.2024.1343795 38414764 PMC10897013

[14] Özkurt E, Hassani MA, Sesiz U. et al. Seed-derived microbial colonization of wild emmer and domesticated bread wheat (*Triticum dicoccoides* and *aestivum*) seedlings shows pronounced differences in overall diversity and composition. *mBio* 2020;11:e02637–20. 10.1128/mBio.02637-20 33203759 PMC7683402

[15] Berendsen RL, Pieterse CMJ, Bakker PAHM. The rhizosphere microbiome and plant health. *Trends Plant Sci* 2012;17:478–86. 10.1016/j.tplants.2012.04.001 22564542

[16] Kloepper J, Schroth M. Plant growth-promoting rhizobacteria on radishes. In: Proceedings of the IV international conference on plant pathogenic bacteria, Vol 2. INRA, France: Gilbert-Clarey Tours, 1978;879–82.

[17] Abdelfattah A, Tack AJM, Lobato C. et al. From seed to seed: the role of microbial inheritance in the assembly of the plant microbiome. *Trends Microbiol* 2023;31:346–55. 10.1016/j.tim.2022.10.009 36481186

[18] Newcombe G, Harding A, Ridout M. et al. A hypothetical bottleneck in the plant microbiome. *Front Microbiol* 2018;9:1645. 10.3389/fmicb.2018.01645 30108556 PMC6080073

[19] Bechtel DB, Abecassis J, Shewry PR. et al. Properties of the wheat grain. *Wheat Chem Technol* 2009;35:51–95. 10.1094/9781891127557.003

[20] Dutta B, Ha Y, Lessl JT. et al. Pathways of bacterial invasion and watermelon seed infection by *Acidovorax citrulli*. *Plant Pathol* 2015;64:537–44. 10.1111/ppa.12307

[21] Barret M, Briand M, Bonneau S. et al. Emergence shapes the structure of the seed microbiota. *Appl Environ Microbiol* 2015;81:1257–66. 10.1128/AEM.03722-14 25501471 PMC4309697

[22] Truyens S, Weyens N, Cuypers A. et al. Bacterial seed endophytes: genera, vertical transmission and interaction with plants. *Environ Microbiol Rep* 2015;7:40–50. 10.1111/1758-2229.12181

[23] Sinnesael A, Eeckhout S, Janssens SB. et al. Detection of *Burkholderia* in the seeds of *Psychotria punctata* (Rubiaceae)—microscopic evidence for vertical transmission in the leaf nodule symbiosis. *PLoS One* 2018;13:1–15. 10.1371/journal.pone.0209091 PMC6294375 30550604

[24] Darrasse A, Barret M, Cesbron S. et al. Niches and routes of transmission of *Xanthomonas citri* pv. fuscans to bean seeds. *Plant Soil* 2018;422:115–28. 10.1007/s11104-017-3329-3

[25] Torres-Cortés G, Genthon C, Briand M. et al. Functional microbial features driving community assembly during seed germination and emergence. *Front Plant Sci* 2018;9:1–16. 10.3389/fpls.2018.00902 30008730 PMC6034153

[26] Chen L, Bao H, Yang J. et al. Dynamics of rice seed-borne bacteria from acquisition to seedling colonization. *Microbiome* 2024;12:253. 10.1186/s40168-024-01978-8 39627882 PMC11613804

[27] Johnston-Monje D, Gutiérrez JP, Lopez-Lavalle LAB. Seed-transmitted bacteria and fungi dominate juvenile plant microbiomes. *Front Microbiol* 2021;12:737616. 10.3389/fmicb.2021.737616 34745040 PMC8569520

[28] Rodríguez CE, Antonielli L, Mitter B. et al. Heritability and functional importance of the *Setaria Viridis* bacterial seed microbiome. *Phytobiomes J* 2020;4:40–52. 10.1094/PBIOMES-04-19-0023-R

[29] Rezki S, Campion C, Simoneau P. et al. Assembly of seed-associated microbial communities within and across successive plant generations. *Plant Soil* 2018;422:67–79. 10.1007/s11104-017-3451-2

[30] Kim H, Jeon J, Lee KK. et al. Longitudinal transmission of bacterial and fungal communities from seed to seed in rice. *Commun Biol* 2022;5:772. 10.1038/s42003-022-03726-w 35915150 PMC9343636

[31] Vannier N, Mony C, Bittebiere AK. et al. A microorganisms’ journey between plant generations. *Microbiome* 2018;6:79. 10.1186/s40168-018-0459-7 29695286 PMC5918900

[32] Bergna A, Cernava T, Rändler M. et al. Tomato seeds preferably transmit plant beneficial endophytes. *Phytobiomes J* 2018;2:183–93. 10.1094/PBIOMES-06-18-0029-R

[33] Sulesky-Grieb A, Simonin M, Bintarti AF. et al. Stable, multigenerational transmission of the bean seed microbiome despite abiotic stress. *mSystems* 2024;9:e00951–24. 10.1128/msystems.00951-24 PMC11575401 39475253

[34] Mitter B, Pfaffenbichler N, Flavell R. et al. A new approach to modify plant microbiomes and traits by introducing beneficial bacteria at flowering into progeny seeds. *Front Microbiol* 2017;8:11. 10.3389/fmicb.2017.00011 28167932 PMC5253360

[35] Compant S, Kaplan H, Sessitsch A. et al. Endophytic colonization of *Vitis vinifera* L. by *Burkholderia phytofirmans* strain PsJN: from the rhizosphere to inflorescence tissues. *FEMS Microbiol Ecol* 2008;63:84–93. 10.1111/j.1574-6941.2007.00410.x 18081592

[36] García-Suárez R, Verduzco-Rosas LA, Del Rincón-Castro MC. et al. Translocation of *Bacillus thuringiensis* in *Phaseolus vulgaris* tissues and vertical transmission in *Arabidopsis thaliana*. *Appl Microbiol Int* 2016;38:42–9. 10.1111/jam.13407 28129468

[37] de Sousa T, Ribeiro M, Sabenca C. et al. The 10,000-year success story of wheat! *Foods* 2021;10:2124. 10.3390/foods10092124 34574233 PMC8467621

[38] García-Rodríguez FM, Toro N. *Sinorhizobium meliloti nfe* (nodulation formation efficiency) genes exhibit temporal and spatial expression patterns similar to those of genes involved in symbiotic nitrogen fixation. *Mol Plant-Microbe Interact* 2000;13:583–91. 10.1094/MPMI.2000.13.6.583 10830257

[39] Garrido-Sanz D, Keel C. Seed-borne bacteria drive wheat rhizosphere microbiome assembly via niche partitioning and facilitation. *Nat Microbiol* 2025;10:1130–44. 10.1038/s41564-025-01973-1 40140705 PMC12055584

[40] Chesneau G, Laroche B, Préveaux A. et al. Single seed microbiota: assembly and transmission from parent plant to seedling. *mBio* 2022;13:e0164822. 10.1128/mbio.01648-22 36222511 PMC9765463

[41] Links MG, Demeke T, Gräfenhan T. et al. Simultaneous profiling of seed-associated bacteria and fungi reveals antagonistic interactions between microorganisms within a shared epiphytic microbiome on *Triticum* and *Brassica* seeds. *New Phytol* 2014;202:542–53. 10.1111/nph.12693 24444052 PMC4235306

[42] Robinson RJ, Fraaije BA, Clark IM. et al. Endophytic bacterial community composition in wheat (*Triticum aestivum*) is determined by plant tissue type, developmental stage and soil nutrient availability. *Plant Soil* 2016;405:381–96. 10.1007/s11104-015-2495-4

[43] Abdullaeva Y, Ratering S, Ambika Manirajan B. et al. Domestication impacts the wheat-associated microbiota and the rhizosphere colonization by seed- and soil-originated microbiomes, across different fields. *Front Plant Sci* 2022;12:8069f15. 10.3389/fpls.2021.806915 PMC8789879 35095978

[44] Simonin M, Briand M, Chesneau G. et al. Seed microbiota revealed by a large-scale meta-analysis including 50 plant species. *New Phytol* 2022;234:1448–63. 10.1111/nph.18037 35175621

[45] Duchateau S, Crouzet J, Dorey S. et al. The plant-associated *Pantoea* spp. as biocontrol agents: mechanisms and diversity of bacteria-produced metabolites as a prospective tool for plant protection. *Biol Control* 2024;188:105441. 10.1016/j.biocontrol.2024.105441

[46] Walterson AM, Stavrinides J. *Pantoea*: insights into a highly versatile and diverse genus within the Enterobacteriaceae. *FEMS Microbiol Rev* 2015;39:968–84. 10.1093/femsre/fuv027 26109597

[47] Scala V, Faino L, Costantini F. et al. Analysis of Italian isolates of *Pantoea stewartii* subsp. stewartii and development of a real-time PCR-based diagnostic method. *Front Microbiol* 2023;14:105441. 10.3389/fmicb.2023.1129229 PMC10174441 37180265

[48] Kioroglou D, Mas A, del Carmen M. Evaluating the effect of QIIME balanced default parameters on metataxonomic analysis workflows with a mock community. *Front Microbiol* 2019;10:1084. 10.3389/fmicb.2019.01084 31156593 PMC6532570

[49] Cregger MA, Veach AM, Yang ZK. et al. The *Populus* holobiont: dissecting the effects of plant niches and genotype on the microbiome. *Microbiome* 2018;6:31. 10.1186/s40168-018-0413-8 29433554 PMC5810025

[50] Hayes RA, Rebolleda-Gómez M, Butela K. et al. Spatially explicit depiction of a floral epiphytic bacterial community reveals role for environmental filtering within petals. *Microbiology open* 2021;10:e1158. 10.1002/mbo3.1158 33650801 PMC7859501

[51] Wassermann B, Müller H, Berg G. An apple a day: which bacteria do we eat with organic and conventional apples? *Front Microbiol* 2019;10:1629. 10.3389/fmicb.2019.01629 31396172 PMC6667679

[52] Tkacz A, Pini F, Turner TR. et al. Agricultural selection of wheat has been shaped by plant-microbe interactions. *Front Microbiol* 2020;11:132. 10.3389/fmicb.2020.00132 32117153 PMC7015950

[53] Gonella E, Orrù B, Marasco R. et al. Disruption of host-symbiont associations for the symbiotic control and management of Pentatomid agricultural pests—a Review. *Front Microbiol* 2020;11:547031. 10.3389/fmicb.2020.547031 33329418 PMC7728854

[54] Schlaeppi K, Bulgarelli D. The plant microbiome at work. *Mol Plant-Microbe Interact* 2014;28:212–7. 10.1094/MPMI-10-14-0334-FI 25514681

[55] Li T, Wu S, Yang W. et al. How Mycorrhizal associations influence orchid distribution and population dynamics. *Front Plant Sci* 2021;12:647114. 10.3389/fpls.2021.647114 34025695 PMC8138319

[56] Compant S, Samad A, Faist H. et al. A review on the plant microbiome: ecology, functions, and emerging trends in microbial application. *J Adv Res* 2019;19:29–37. 10.1016/j.jare.2019.03.004 31341667 PMC6630030

[57] Sharma P, Pandey R, Chauhan NS. Unveiling wheat growth promotion potential of phosphate solubilizing *Pantoea agglomerans* PS1 and PS2 through genomic, physiological, and metagenomic characterizations. *Front Microbiol* 2024;15:1467082. 10.3389/fmicb.2024.1467082 39318437 PMC11420927

[58] Shariati VJ, Malboobi MA, Tabrizi Z. et al. Comprehensive genomic analysis of a plant growth-promoting rhizobacterium *Pantoea agglomerans* strain P5. *Sci Rep* 2017;7:15610. 10.1038/s41598-017-15820-9 29142289 PMC5688152

[59] Rodríguez CE, Mitter B, Antonielli L. et al. Roots and panicles of the C4 model grasses *Setaria Viridis* (L). and *S. Pumila* host distinct bacterial assemblages with core taxa conserved across host genotypes and sampling sites. *Front Microbiol* 2018;9:2708. 10.3389/fmicb.2018.02708 30483233 PMC6240606

[60] Valles-Colomer M, Blanco-Míguez A, Manghi P. et al. The person-to-person transmission landscape of the gut and oral microbiomes. *Nature* 2023;614:125–35. 10.1038/s41586-022-05620-1 36653448 PMC9892008

[61] Miller I . Bacterial leaf nodule Symbiosis. In: Callow J.A. (ed.), Advance Botanical Research. Amsterdam: Academic Press, 2001, 163–234.

[62] Frank AC, Saldierna Guzmán JP, Shay JE. Transmission of bacterial endophytes. *Microorganisms* 2017;5:70. 10.3390/microorganisms5040070 29125552 PMC5748579

[63] Vandenkoornhuyse P, Quaiser A, Duhamel M. et al. The importance of the microbiome of the plant holobiont. *New Phytol* 2015;206:1196–206. 10.1111/nph.13312 25655016

