# Supplementary material for: Seed-mediated vertical transmission of Pantoea core endophytes
Source: ISME J. 2025 Aug 28;19(1):wraf192. doi: 10.1093/ismejo/wraf192 (PMC12448475; doi:10.1093/ismejo/wraf192)
Supplement: Supplementary_materials_wraf192 [file supplementary_materials_wraf192.pdf]

SUPPLEMENTAL MATERIAL

Seed-mediated vertical transmission of *Pantoea* core endophytes

Irene Sanz-Puente[1], Santiago Redondo-Salvo[1,2], Gloria Torres-Cortés [3¥],

María de Toro[4], Susana Fernandes[5], Andreas Börner[6], Óscar Lorenzo[5],

Fernando de la Cruz[1*], and Marta Robledo[1,2*]

[1] *Instituto de Biomedicina y Biotecnología de Cantabria (IBBTEC), Universidad de Cantabria - Consejo Superior de Investigaciones Científicas (CSIC), Santander, Spain.*

[2] *Biomar Microbial Technologies, Parque Tecnológico de León, Armunia, León, Spain*

[3] *Institut Agro, INRAE, IRHS, Université d'Angers, Angers, France.*

[4] *Genomics and Bioinformatics Core Facility, Center for Biomedical Research of La Rioja, Logroño, Spain.*

[5] *Departamento de Botánica y Fisiología Vegetal, Instituto de Investigación en Agrobiotecnología (CIALE), Universidad de Salamanca, Salamanca, Spain*

[6] *Genebank Department, Leibniz Institute of Plant Genetics and Crop Plant Research (IPK), Seeland/OT, Gatersleben, Germany*

[¥] *Present address: Innoplant S.L, Avenida Alfaguara 62, Alfacar, Granada, Spain*

* For correspondence: Dr. Marta Robledo, marta.robledo@unican.es, or Prof. Fernando de la Cruz: delacruz@unican.es

Instituto de Biomedicina y Biotecnología de Cantabria (IBBTEC), Universidad de Cantabria - CSIC, C/ Albert Einstein 22, Santander, Cantabria, 39011, Spain.

This document contains supplemental figures and tables for the indicated manuscript

Total number of pages of Supporting Information: 16 (including cover page)

Number of Figures in Supporting Information: 3

Number of Tables in Supporting Information: 6

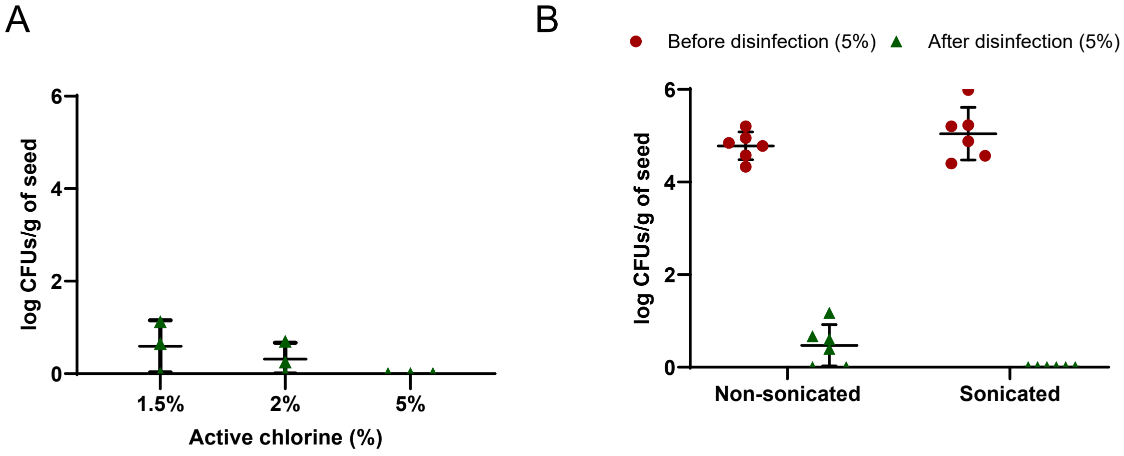

Figure S1. Optimization of wheat seed endophytic microbiome analysis. A) Comparison of three disinfection methods of sonicated wheat seeds, presented as CFUs per seed gram after disinfection with three different active chloride concentrations: 1.5% (Robinson et al., 2016); 2% (Torres-Cortés et al., 2018); and 5% (adapted from Mitter et al., 2017). Each point represents a measure from three independent assays (with 3 pools of 12 seeds per replicate). Error bars indicate SD. B) Bacterial load in non-sonicated and sonicated pooled samples of wheat (*Triticum aestivum* var Craklin) seeds. Colony Forming Units (CFUs) were measured before (red points) or after (green points) surface disinfection with 5% active chlorine. Each point represents a technical replicate from each of the two independent assays (each biological replicate is a pool of 12 seeds). Error bars indicate SD.

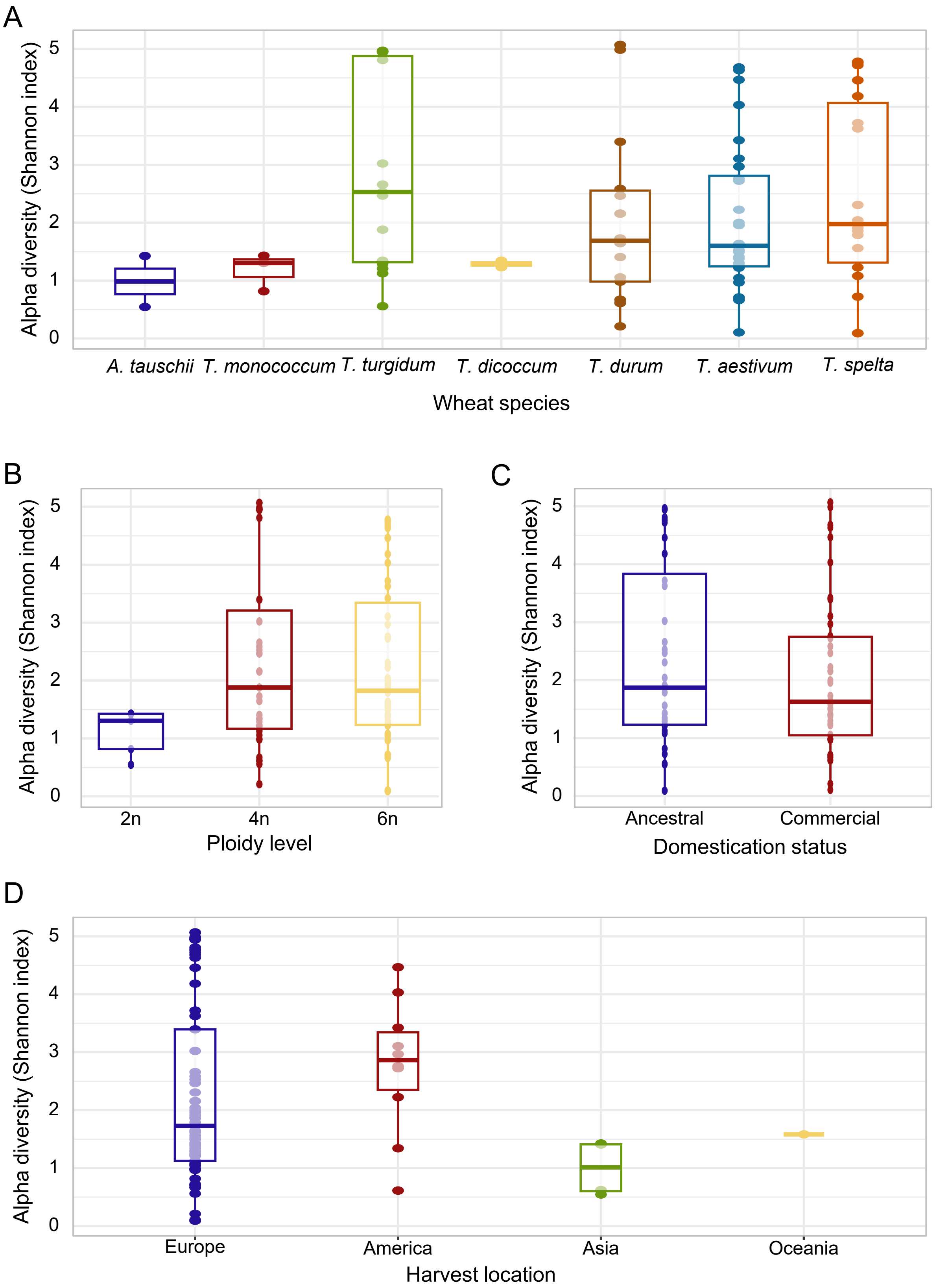

Figure S2. Analysis of wheat seed bacterial worldwide alpha diversity. Box-and-whisker plots show α-diversity metrics (Shannon index) of 24 seed samples (each point is the average of 12 seeds). Samples were grouped according to wheat species (A), ploidy level (B), domestication level (C) or known harvest location (D) as indicated in the different panels. Each boxplots show the distribution of Shannon diversity index across wheat species, with the median, interquartile range (IQR), and potential outliers.

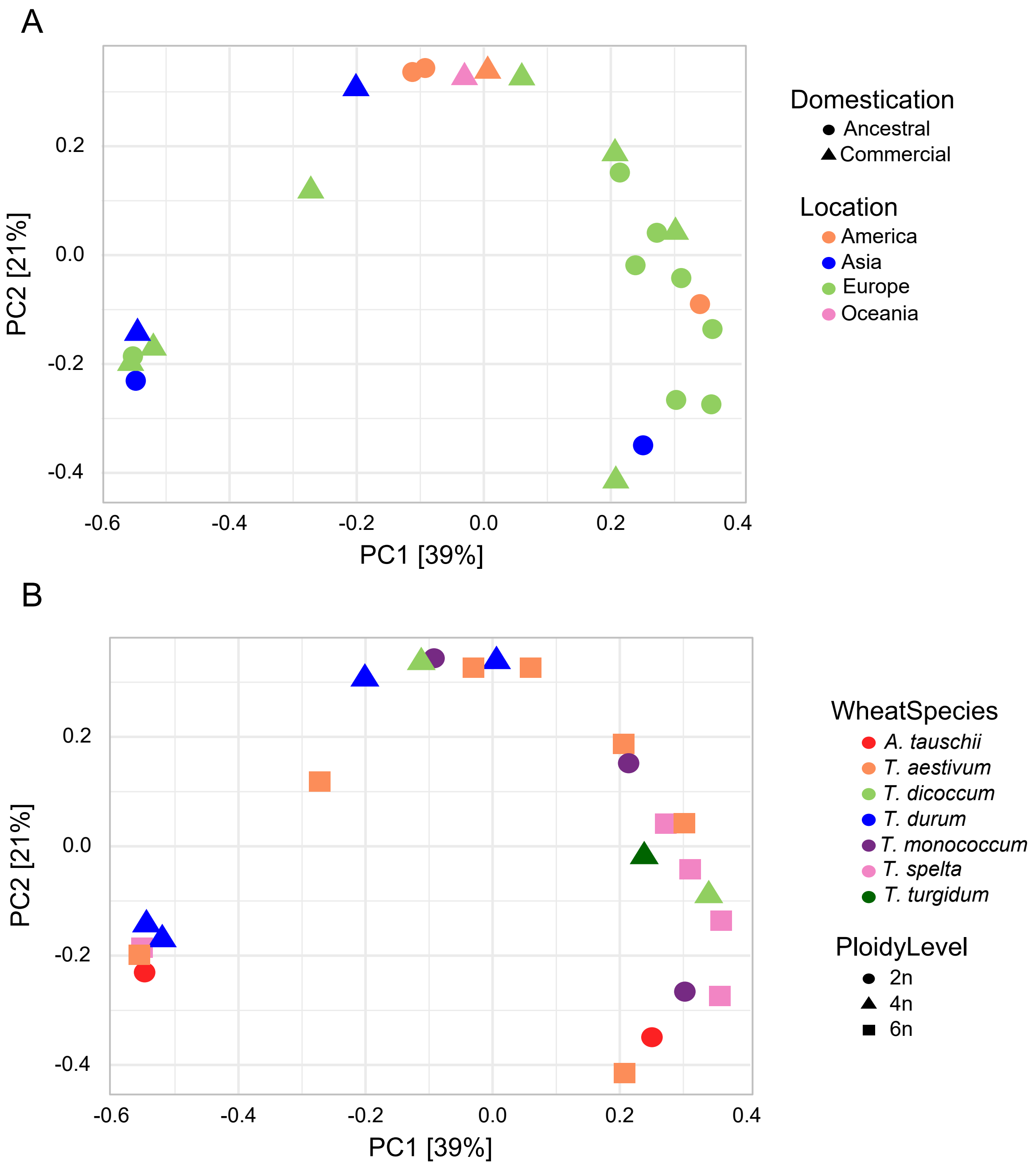

Figure S3. Analysis of wheat seed bacterial worldwide structure. PCoA corresponding to the Bray–Curtis dissimilarity index (β-diversity) of the bacterial communities present in wheat seeds depicted according to the legends. Each dot represents an individual technical replicate. The x- and y-axes represent the first and second components of the PCoA plot, respectively. Similarly, Bray–Curtis PERMANOVA revealed that bacterial seed communities did not significantly differ based on host wheat species ($R^2 = 0.27$), polyploidy level ($R^2 = 0.05$), known harvest location ($R^2 = 0.18$), or domestication status ($R^2 = 0.07$).

Table S1. Wheat samples used in this study that were harvested in Spain (_SP) from two different fields located in Asturias or Leon. Samples in bold were used in figures 3 and 4 (see Table S3). [a]Genotypes resulting from hybridizations and cultivated species: AA *T. monococcum* from *T. urartu*, AABB *T. turgidum,* and *T. durum* from the hybridization with *Aegilops speltoides* (BB), AABBDD *T. aestivum* and *T.spelta*.

<table>
<tr><th>Sample</th><th>Specie</th><th>Location</th><th>Harvest Year</th><th>[a]Genome</th><th>Taming</th></tr>
<tr><td>Tmo_SP</td><td>T. monococcum</td><td>Asturias</td><td>2020</td><td>AA</td><td rowspan="2">Ancestral</td></tr>
<tr><td>Ttu_SP</td><td>T. turgidum</td><td>Asturias</td><td>2019</td><td rowspan="2">AABB</td></tr>
<tr><td>Tdu_SP</td><td>T. durum</td><td rowspan="5">Leon</td><td rowspan="2">2018</td><td rowspan="5">Commercial</td></tr>
<tr><td>Tae_SP1</td><td rowspan="4">T. aestivum</td><td rowspan="9">AABBDD</td></tr>
<tr><td>Tae_SP2</td><td rowspan="2">2019</td></tr>
<tr><td>Tae_SP4</td></tr>
<tr><td>Tae_SP5</td><td>2020</td></tr>
<tr><td>Tpe_SP1</td><td rowspan="5">T. spelta</td><td rowspan="4">Asturias</td><td rowspan="4">2019</td><td rowspan="5">Ancestral</td></tr>
<tr><td>Tpe_SP2</td></tr>
<tr><td>Tpe_SP3</td></tr>
<tr><td>Tpe_SP4</td></tr>
<tr><td>Tpe_SP5</td><td>Leon</td><td>2020</td></tr>
</table>

Table S2. Wheat seed samples used in this study that belong to the seedbank from the Institute of Plant Genetics and Crop Plant Research (IPK). The collection includes four different *Triticum* species and *Aegilops tauschii*, one of the three progenitors of the hexaploid wheat [1,2].[a]Genotypes resulting from hybridizations and cultivated species: DD *A. tauschii*, AA *T. monococcum* from *T. urartu*, AABB *T. dicoccom* and *T. durum* from the hybridization with *Aegilops speltoides* (BB), AABBDD *T. aestivum*.

<table>
<tr><th>Sample</th><th>IPK Code</th><th>Genus</th><th>Specie</th><th>Country</th><th>Location</th><th>Year</th><th>[a]Genome</th><th>Taming</th></tr>
<tr><td>Ata_RU</td><td>AE498</td><td rowspan="2">Aegilops</td><td rowspan="2">tauschii</td><td>Russia</td><td rowspan="2">Asia</td><td>1979</td><td rowspan="2">DD</td><td rowspan="6">Ancestral</td></tr>
<tr><td>Ata_UZ</td><td>AE246</td><td>Uzbekistan</td><td>1976</td></tr>
<tr><td>Tmo_BU</td><td>TRI1998</td><td rowspan="10">Triticum</td><td rowspan="2">monococcum</td><td>Bulgaria</td><td>Europe</td><td rowspan="7">1975</td><td rowspan="2">AA</td></tr>
<tr><td>Tmo_UN</td><td>TRI4323</td><td rowspan="2">Unknown</td><td rowspan="2">Unknown</td></tr>
<tr><td>Tdi_UN</td><td>TRI4168</td><td rowspan="2">dicoccum</td><td rowspan="5">AABB</td></tr>
<tr><td>Tdi_US</td><td>TRI445</td><td>United States</td><td rowspan="2">America</td></tr>
<tr><td>Tdu_AR</td><td>TRI448</td><td rowspan="3">durum</td><td>Argentina</td><td rowspan="6">Commercial</td></tr>
<tr><td>Tdu_AF</td><td>TRI4101</td><td>Afghanistan</td><td rowspan="2">Asia</td></tr>
<tr><td>Tdu_IR</td><td>TRI6263</td><td>Iran</td></tr>
<tr><td>Tae_GE</td><td>TKS</td><td rowspan="3">aestivum</td><td>Germany</td><td rowspan="2">Europe</td><td>2016</td><td rowspan="3">AABBDD</td></tr>
<tr><td>Tae_GR</td><td>TRI10765</td><td>Greece</td><td rowspan="2">1975</td></tr>
<tr><td>Tae_NZ</td><td>TRI7121</td><td>New Zealand</td><td>Oceania</td></tr>
</table>

Table S3. Plant and soil field samples used in this study. All samples were harvested in Leon, Spain.

Sample	Species	Tissue	[a]Genome	Taming
Tdu_So		soil		
Tdu_Ro		root		
Tdu_Sh	*T. durum*	shoot		Commercial
Tdu_Pk		spike		
Tdu_Se		seed	AABB	
Ttu_So		soil		
Ttu_Ro		root		
Ttu_Sh	*T. turgidum*	shoot		
Ttu_Pk		spike		
Ttu_Se		seed		Ancestral
Tpe_So		soil		
Tpe_Ro		root		
Tpe_Sh	*T. spelta*	shoot		
Tpe_Pk		spike		
Tpe_Se		seed	AABBDD	
Tae_So		soil		
Tae_Ro		root		
Tae_Sh	*T. aestivum*	shoot		Commercial
Tae_Pk		spike		
Tae_Se		seed		

Table S4. Primers used in this study. *with the corresponding Illumina adapters when required.

Primer code	Sequence	Reference
27_F	5'-AGAGTTTGATCMTGGCTCAG-3'	[3]
515_F*	5’-GTGCCAGCMGCCGCGGTAA-3’	[4]
808_R*	5’-GACTACHVGGGTATCTAATCC-3’	[5]
1522_R	5'-AAGGAGGTGATCCANCCRCA-3'	[6]
GUS_F	5'-GTTCATAGAGATAACCT-3'	[7]
GUS_R	5'-TTAGCTCACTCATTAGG-3'	[7]
gusA_R	5'-TGGTGTAGAGCATTACGCTGCGAT-3'	This work
gusA_F	5'-ACTCATTACGGCAAAGTGTGGGTCA-3'	This work
gusA-FAM	5'-AGCATCAGGGCGGCTATACGCC-3'	This work

Table S5. SNPs found between *P. agglomerans* strains C-113 and C-204 genomes. *IGR: Intergenic region.

Contig	position	Change	Gene*	Product	Effect
AMHFCLMC_3	164621	A to G	IGR	98 bp upstream hypothetical protein AMHFCLMC_01984	Unknown
AMHFCLMC_5	105621	T to C	IGR	346 bp upstream hypothetical protein AMHFCLMC_02771	Unknown
AMHFCLMC_13	88215	A to G	*cytR*	HTH-type transcriptional repressor CytR	Stop codon elimination

Table S6. Gene annotations identified in *Pantoea agglomerans* strain C113 genome potentially involved in plant growth promotion.

Biological task	locus_tag	gene	EC_ number	COG	Product
Phosphate transport and solubilization	AMHFCLMC _04414	*pstC*		COG0573	Phosphate transport system permease protein PstC
	AMHFCLMC _04415	*pstA*		COG0581	Phosphate transport system permease protein PstA
	AMHFCLMC _04416	*pstB*	7.3.2.1	COG1117	Phosphate import ATP-binding protein PstB
	AMHFCLMC _04417	*phoU*		COG0704	Phosphate-specific transport system accessory protein PhoU
	AMHFCLMC _00143	*phnF*		COG2188	putative transcriptional regulator PhnF

	AMHFCLMC _00144	*phnG*	2.7.8.37	COG3624	Alpha-D-ribose 1-methylphosphonate 5-triphosphate synthase subunit
	AMHFCLMC _00145	*phnH*	2.7.8.37	COG3625	Alpha-D-ribose 1-methylphosphonate 5-triphosphate synthase subunit
	AMHFCLMC _00146	*phnI*	2.7.8.37	COG3626	Alpha-D-ribose 1-methylphosphonate 5-triphosphate synthase subunit
	AMHFCLMC _00147	*phnJ*	4.7.1.1	COG3627	Alpha-D-ribose 1-methylphosphonate 5-phosphate C-P lyase
	AMHFCLMC _00148	*phnK*		COG4107	Putative phosphonates utilization ATP-binding protein PhnK
	AMHFCLMC _00149	*phnL*	2.7.8.37	COG4778	Alpha-D-ribose 1-methylphosphonate 5-triphosphate synthase subunit
	AMHFCLMC _00150	*phnM*	3.6.1.63	COG3454	Alpha-D-ribose 1-methylphosphonate 5-triphosphate diphosphatase
	AMHFCLMC _00151	*phnN*	2.7.4.23	COG3709	Ribose 1,5-bisphosphate phosphokinase PhnN
	AMHFCLMC _00152	*phnP*	3.1.4.55	COG1235	Phosphoribosyl 1,2-cyclic phosphate phosphodiesterase
	AMHFCLMC _00153	*phnC*	7.3.2.1	COG3638	Phosphate-import ATP-binding protein PhnC
	AMHFCLMC _00154	*phnD*		COG3221	Phosphate-import protein PhnD
IAA Biosynthesis	AMHFCLMC _02585	*iaaH*	3.5.1.-		Indole-3-acetyl-aspartic acid hydrolase
	AMHFCLMC _02998	*ipdC*	4.1.1.74	COG3961	Indole-3-pyruvate decarboxylase
Siderophore biogenesis	AMHFCLMC _02485	*entF*	2.7.7.-	COG1020	Enterobactin synthase component F
	AMHFCLMC _02486	*fepC*		COG1120	Ferric enterobactin transport ATP-binding protein FepC
	AMHFCLMC _02487	*fepG*		COG4779	Ferric enterobactin transport system permease protein FepG
	AMHFCLMC _02488	*fepD*		COG0609	Ferric enterobactin transport system permease protein FepD
	AMHFCLMC _02489	*entS_3*		COG0477	Enterobactin exporter EntS
	AMHFCLMC _02490	*fepB*		COG4592	Ferrienterobactin-binding periplasmic protein
	AMHFCLMC _02491	*entC*	5.4.4.2	COG1169	Isochorismate synthase EntC
	AMHFCLMC _02492	*entE*	6.3.2.14	COG1021	Enterobactin synthase component E

	AMHFCLMC_02493	*entB*	6.3.2.14	COG1535	Enterobactin synthase component B
	AMHFCLMC_02494	*entA*	1.3.1.28	COG1028	2,3-dihydro-2,3-dihydroxybenzoate dehydrogenase
Bacterial adhesion	AMHFCLMC_03403	*pilQ*			Type IV pilus biogenesis and competence protein PilQ
	AMHFCLMC_02180	*fimA*			Type-1 fimbrial protein, A chain
GABA metabolism	AMHFCLMC_00109	*gabD*	1.2.1.79	COG1012	Succinate-semialdehyde dehydrogenase [NADP(+)]
	AMHFCLMC_01924	*gabR_1*		COG1167	HTH-type transcriptional regulatory protein GabR
	AMHFCLMC_01926	*gabP*		COG1113	GABA permease
Volatile organic compounds synthesis	AMHFCLMC_03838	*budC*	1.1.1.304		Diacetyl reductase [(S)-acetoin forming]
	AMHFCLMC_04006	*ilvI*	2.2.1.6	COG0028	Acetolactate synthase isozyme 3 large subunit
	AMHFCLMC_04007	*ilvH*	2.2.1.6	COG0440	Acetolactate synthase isozyme 3 small subunit
Polyamine biosynthesis	AMHFCLMC_02808	*speA*	4.1.1.19	COG1166	Biosynthetic arginine decarboxylase
	AMHFCLMC_02809	*speB*	3.5.3.11	COG0010	Agmatinase
	AMHFCLMC_04070	*speD*	4.1.1.50	COG1586	S-adenosylmethionine decarboxylase proenzyme
	AMHFCLMC_04071	*speE*	2.5.1.-	COG0421	Polyamine aminopropyltransferase
	AMHFCLMC_00602	*potA_1*	7.6.2.11		Spermidine/putrescine import ATP-binding protein PotA

EXTENDED MATERIAL AND METHODS

Sample collection

The endophytic bacterial communities of wheat analyzed in Figure 1 were characterized using 24 seed samples from two main sources (Table S1-S3). In Spain, samples were collected from five major wheat-growing regions: two plots in Leon (Matanza and Villaornate), one plot in Valladolid (Melgar de Abajo) and two plots in Asturias (Villaviciosa and Quinzanas). The wheat species were categorized into commercial and ancestral types (Table S1) based on their genome content and approximate divergence times, before and after 0.13 million years ago, respectively[2].

An additional set of seed samples (Table S2), originating from various global locations, were regenerated at the Leibniz Institute of Plant Genetics and Crop Plant Research (IPK) germplasm bank in Gatersleben, Germany.

Senescent plants (Table S3) were harvested in Leon (Spain) at the BBCH 92 growth stage, when grains are fully mature and dry enough for storage without further drying. Rhizospheric soil surrounding wheat roots was also collected in sterile tubes. Sampling was conducted directly from cultivated plots, with three sampling sites assessed per location. Approximately ten individual wheat plants were collected per plot. The plants were placed in paper bags and transported immediately to the laboratory, where they were stored at 4°C until further processing.

Consecutive generations of *T. aestivum* var. Chambo seeds used for *Pantoea* isolation and whole-genome sequencing correspond to samples Tae_SP1, Tae_SP2, and Tae_SP4 (Table S1) from the initial profiling effort.

For the greenhouse experiments, seeds from *T. aestivum* (Tae_SP4, Table S1), *Lolium multiflorum* var. grazing, and the universal model plant *Arabidopsis thaliana* ecotype Columbia-0 (Col-0) were used.

Plant surface disinfection

From field locations, one representative wheat plant per sampling site was selected, resulting in three plants per location. In the laboratory, each plant was dissected using sterile scalpels into four tissue types: (1) roots, consisting of subsurface material with visible fine roots; (2) shoots, defined as the above-ground stem tissue between the second and third fully expanded leaves, approximately 15 cm in length; (3) spikes, referring to the unbranched inflorescence structure; and (4) seeds, represented by mature grains (endosperms) collected directly from the harvested spikes. Aproximately 0.5 g of each plant tissue per individual plant was transferred into a sterile 1.5 ml microtube tuve containing 1 mL PBST (Phosphate Buffered Saline with 0.05% Tween-20). Samples were then sonicated for 1 min (Ultrasons, Barcelona, Spain).

Three different disinfection protocols mentioned in previous literature were tested on wheat seeds: 1) Seeds were rinsed in 1 mL 70% ethanol for 3 min, followed by 1 ml 5% of NaHClO (Active Chlorine) for 5 min [8]. 2) In 1 ml 70% ethanol for 10 min, 1.5% for NaHClO 1 h at 4°C [9]. 3) In 96% ethanol 1 min, 2% of NaHClO for 5 min, and two additional washes of 96% ethanol 30 secs, [10].Wheat seed sonication followed by the surface disinfection method described by Mitter et al. in 2017 were routinely performed prior to further analysis.

Wheat roots were surface-disinfected by rinsing in 1 mL of 70% ethanol for 5 min, followed by 1 mL of 5% sodium hypochlorite (NaOCl) for 10 min. Wheat spikes and shoots were treated similarly but rinsed in 1 mL of 70% ethanol for 3 min before the NaOCl treatment. All surface-disinfected wheat tissues were subsequently rinsed three times with 1 mL of sterile distilled water (SDW), 1 min per rinse.

Arabidopsis thaliana seeds were disinfected by immersion in 1 mL of 3.5% bleach supplemented with 0.01% Triton X-100 for 5 min at room temperature. After centrifugation, the supernatant was discarded, and seeds were washed four times with 1 mL of SDW, 1 min per wash. *Lolium* seeds underwent a similar procedure but were treated with 1 mL of 3.5% bleach + 0.01% Triton for 8 min, followed by three 1-min rinses in 1 mL of SDW.

To verify the effectiveness of the surface disinfection protocols, 100 µL of the final wash water was plated onto tryptic soy agar (TSA; Condalab, Madrid, Spain). Plates were incubated at 30°C for at least 3 days. Surface-disinfected plant materials were stored at 4°C until further analysis.

Bacterial isolation

To isolate endophytic bacteria, approximately 0.25 g of surface-disinfected plant material was excised into at least two pieces using a sterile scalpel. The tissue was then disrupted and homogenized using a sterile mortar and pestle. Following homogenization, sterile phosphate-buffered saline (PBS) was added at a ratio of ~1 mL per gram of plant material. The resulting suspensions were incubated overnight at 4 °C on a tube rotator (Stuart SB3) set to 60 rpm.

After incubation, PBS suspensions were serially diluted and plated on TSA to isolate endophytic bacteria from the wheat seeds. Plates were incubated in the dark at 30 °C for at least one week. Colonies were subsequently subcultured under the same conditions. Yellow, *Pantoea*-like colonies were selected for further characterization and downstream experiments.

DNA extraction

Genomic DNA from endophytic bacterial isolates obtained from wheat seeds was extracted using the InstaGene Matrix (Bio-Rad, California, USA), following the manufacturer's protocol.

For microbial community profiling, DNA was extracted from surface-disinfected plant tissues and rhizospheric soil samples. Briefly, 0.25 g of surface-sterilized plant material was cut into two or more pieces using a sterile scalpel and homogenized using a FastPrep-24 instrument (MP Biomedicals, California, USA). DNA extraction was performed using the DNeasy PowerLyzer PowerSoil Kit (Qiagen, Hilden, Germany) according to the manufacturer's instructions, with a minor modification: in step five, PowerBead tubes were centrifuged for 3 min at 10,000 × g. Final DNA elution was carried out in 50 µL of pre-warmed Milli-Q water.

DNA concentrations were measured using a NanoDrop spectrophotometer (Thermo Fisher Scientific, Massachusetts, USA), and all samples were stored at –20 °C until further analysis.

Sequencing controls and contaminants removal

All sequencing sample handling was carried out under sterile conditions using DNA-free consumables to minimize the risk of external contamination. To monitor potential contaminants introduced during sample processing and library preparation, we included DNA

extraction blanks, PCR negative controls, and storage bag blanks in the sequencing runs. Putative contaminants were identified and removed from the entire dataset using Decontam package [11], appliying the prevalence-based method with ascore threshold of 0.1 to differentiate true biological signals from background noise.

Analysis of Microbial Communities

Taxonomic composition and diversity were assessed using the Phyloseq R package [12]. A Phyloseq object containing taxonomic assignments and ASVa bundance data was used for creating taxonomic bar plots and calculating both α-diversity and β-diversity. The ggplot2 R package [13] was used to generate bar plots visualizing the relative abundance of taxa across samples.

Alpha-diversity was assessed using the `estimate_richness()` function from the Phyloseq package, with diversity restricted to the Shannon index. Resulting values were assigned to their corresponding sample metadata for subsequent statistical analyses and visualizations. Samples with missing or undefined metadata were excluded from the statistical analysis.

Beta-diversity was assessed based on dissimilarity distance measures (Bray-Curtis) and visualized through Principal Coordinates Analysis (PCoA). Ordination was performed with the `ordinate()` function from the Phyloseq package, and the resulting coordinates were plotted using ggplot2.To visualize patterns in beta-diversity, ellipses representing 95% confidence intervals were added to aid interpretation of group dispersion, using the `stat_ellipse()` function in ggplot2.

To explore overlap patterns among ASVs, the ComplexUpset package was used (Krassowski, M. https://github.com/krassowski/complex-upset, accessed 26 March 2025). Presence/absence matrices were generated from ASV abundance tables derived from previously created Phyloseq objects. These matrices were integrated with taxonomic and sample metadata to classify ASVs by plant tissue, wheat genotype, and species. UpSet plots were then constructed to visualize shared and unique ASVs among sample categories

Abundance-occupancy analysis was performed following the previously described framework [14]. Occupancy was calculated as the proportion of samples in which a given taxon was present, and the average abundance was computed across all samples. These two metrics were used to generate abundance-occupancy distribution plots. Visualizations were created using ggplot2, where each taxon was plotted based on its occupancy and abundance, with a color gradient representing abundance levels. A logarithmic scale was applied to the x-axis to improve visualization clarity.

Statistical Analysis

Prior to statistical testing, the assumptions of normality and homogeneity of variances were evaluated. Normality was assessed for each group using the Shapiro–Wilk test (`shapiro.test()` function), applied via the `by()` function in R. Homogeneity of variances across groups was examined using both Bartlett's test (`bartlett.test()`) and Levene's test (`leveneTest` function from the car package).

If both assumptions were met, one-way analysis of variance (ANOVA; `aov()`) was used to test for significant differences among groups. When ANOVA results were significant ($P < 0.05$), Tukey's Honest Significant Difference (HSD) post hoc test (`TukeyHSD()`) was applied for pairwise comparisons.

In cases where the normality assumption was not satisfied, the non-parametric Kruskal–Wallis test (`kruskal.test()`) was used. If the Kruskal–Wallis test indicated significant differences, Dunn's post hoc test (`dunnTest()` from the FSA package) with Benjamini–Hochberg correction was performed for multiple pairwise comparisons.

For specific pairwise comparisons where normality was not assumed, the Wilcoxon rank-sum test (`wilcox.test()`) was used to determine statistical significance.

To assess the influence of experimental factors on bacterial community composition (Beta-diversity), two PERMANOVA analyses based on Bray–Curtis dissimilarity were conducted using the `adonis()` function from the vegan package. The first model tested all factors simultaneously (Bray–Curtis ~ Location + Ploidy + Domestication + Wheat species), and the second consisted of individual models evaluating each factor independently. All PERMANOVA tests were run with 999 permutations.

A P value < 0.05 was considered statistically significant in all analyses. All statistical procedures were performed in R. The full analysis workflow, including code and data subsets used for specific comparisons, is available in the GitHub repository.

Differential abundance analysis of taxa was performed using the ANCOM-BC2 [15] module provided by QIIME2; taxa with $P < 0.05$ were considered significant.

Compositional differences among plant compartments (Seed, Shoot, Root and Spike) were assessed with Linear Discriminant Analysis (LDA) Effect Size (LEfSe) [16] as implemented in the microbiomeMarker R package [17]. The OTU table at genus level was centred-log-ratio transformed, library-size normalised (`CPM()`) and tested with a Kruskal–Wallis class comparison followed by pair-wise Wilcoxon tests. Features with $P \leq 0.05$ in both tests and LDA scores ≥ 2.0 were retained.

Whole-Genome Sequencing (WGS) and analysis of *Pantoea* seed isolates

To compare the genomes of *Pantoea* seed endophytes, bacterial isolates were obtained from consecutive generations of *T. aestivum var. Chambo* seeds (Tae_SP1, Tae_SP2, and Tae_SP4; Table S1) harvested from a field in León, Spain, as previously described. Yellow colonies were identified via full-length 16S rRNA gene sequencing using primers 27_F, 1522_R, and 515_F (Table S4).

Whole-genome sequencing of *Pantoea* isolates were performed by MicrobesNG (Birmingham, United Kingdom) using Illumina short-read technology. The service provider conducted DNA extraction, library preparation, sequencing, demultiplexing, and initial quality control. Quality of trimmed reads was further assessed using FastQC v0.11.9 [18]. Reads were assembled into contigs using Unicycler v0.5.1 [19]. Genome assembly quality was evaluated with QUAST version 5.3.0 [20].

Complete genome sequences of the three strains were obtained in FASTA format. Strain C113 (PG0) was selected as the reference genome for comparative analyses. Visualization of genomic similarities and differences among the strains was performed using BLAST Ring Image Generator (BRIG) [21], with minimum identity thresholds and color settings adjusted to highlight conserved and divergent genomic regions. The resulting images facilitated a visual assessment of genomic relationships among the analyzed strains.

Single nucleotide polymorphisms (SNPs) were identified using Snippy v4.6.0 [22] with default parameters. Reads were aligned to the reference genome using BWA-MEM v0.7.17. All bioinformatic analyses were conducted in a Linux environment (Ubuntu 20.04).

Gene prediction and functional annotation of protein-coding genes was performed using Prokkaversion 1.14.6 [23], with NCBI-compliant mode enabled to exclude contigs shorter than 200 bp and to allow the identification of non-coding RNAs using Infernal and Rfam databases.

Detection of *Pantoea agglomerans* C88-GUS in Plant Tissues

G1 and G2 seeds were surface-disinfected and germinated as previously described. Seven-day-old seedlings were immersed in GUS staining buffer (50 mM phosphate buffer, 0.05% Triton X-100, 1 mM $K_3Fe(CN)_6$, 1 mM $K_4Fe(CN)_6$, 0.05 M EDTA, and 1.2 mM X-gluc). Samples were incubated at 37°C in the dark until blue coloration appeared, indicating β-glucuronidase activity. Stained seedlings were then washed twice with distilled water, stored in 70% ethanol, and imaged using a stereoscopic microscope (Olympus SZX16/SZ12) with a Nikon DS-Fi1 camera.

To confirm and quantify colonization by the *P. agglomerans* C88-GUS strain, nine seven-day-old seedlings were surface-disinfected and dissected into shoot, root, and remaining seed tissues. Tissues were pooled in triplicates and subjected to DNA extraction as previously described. The presence of the *gusA* gene, encoding β-glucuronidase, was quantified by real-time PCR on a StepOnePlus Real-Time PCR System (ThermoFisher). Each 20 µL reaction consisted of 4 µL PerfeCTa qPCR ToughMix, 5 µL DNA, 0.6 µL primers (100 nM), and 0.2 µL TaqMan probe targeting *gusA* (Table S4).

PCR conditions included an initial denaturation at 95°C for 10 min, followed by 40 cycles of 95°C for 10 seconds and 62°C for 40 seconds. All reactions were run in triplicate. Fluorescence data were collected and analyzed using StepOnePlus software. Relative quantification was conducted using a standard curve correlating Ct values with CFU/g of C88-GUS in mock-inoculated plant tissues. CFU values were obtained by mixing known concentrations of *P. agglomerans* C88-GUS with a constant amount of sterile plant material, followed by plating and colony counting. Negative controls (no DNA template and PBS-treated plants) were included in all reactions and consistently yielded Ct values >34 or no detectable signal.

REFERENCES

1.Gaut BS, Seymour DK, Liu Q *et al.* Demography and its effects on genomic variation in crop domestication. *Nat Plants* 2018;**4**:512–20.

2.Gholizadeh S, Mohammadi SA, Salekdeh GH. Changes in root microbiome during wheat evolution. *BMC Microbiol* 2022;**22**:1–17.

3.Lane DJ. 16S/23S rRNA sequencing. In: Stackebrandt, E. and Goodfellow, M. (eds.), Nucleic Acid Techniques in Bacterial Systematics. West Sussex: John Wiley & Sons Ltd., 1991, 115-147.

4.Marchesi JR, Wade JR, Sato T *et al.* Design and evaluation of useful bacterium-specific PCR primers that amplify genes coding for bacterial 16S rRNA. *Appl Environ Microbiol* 1998;**64**:795–9.

5.Caporaso JG, Kuczynski J, Stombaugh J *et al.* QIIME allows analysis of high-throughput community sequencing data. *Nat Methods* 2010;**7**:335–6.

6.Weisburg WG, Barns SM, Pelletier DA *et al.* 16S ribosomal DNA amplification for phylogenetic study. *J Bacteriol* 1991;**173**:697–703.

7.Almasi MA, Aghapour-Ojaghkandi M, Bagheri K *et al.* Comparison and Evaluation of Two Diagnostic Methods for Detection of *npt* II and GUS Genes in *Nicotiana tabacum*. *Appl Biochem Biotechnol* 2015;**175**:3599–616.

8.Mitter B, Pfaffenbichler N, Flavell R *et al.* A new approach to modify plant microbiomes and traits by introducing beneficial bacteria at flowering into progeny seeds. *Front Microbiol* 2017;**8**:1–10.

9.Robinson RJ, Fraaije BA, Clark IM *et al.* Wheat seed embryo excision enables the creation of axenic seedlings and Koch's postulates testing of putative bacterial endophytes. *Sci Rep* 2016;**6**:1–9.

10.Torres-Cortés G, Genthon C, Briand M *et al.* Functional Microbial Features Driving Community Assembly During Seed Germination and Emergence. *Front Plant Sci* 2018;**9**:1–16.

11.Davis NM, Proctor DiM, Holmes SP *et al.* Simple statistical identification and removal of contaminant sequences in marker-gene and metagenomics data. *Microbiome* 2018;**6**:1–14.

12.Mcmurdie PJ, Holmes S. phyloseq: An R Package for Reproducible Interactive Analysis and Graphics of Microbiome Census Data. *PLoS ONE*. 2013;**8(4):**e61217.

13.Wickham H, Winston C, Henry L *et al.* ggplot2: Elegant Graphics for Data Analysis. *Biometrics,* 2011;**67**:678-679

14.Shade A, Stopnisek N. Abundance-occupancy distributions to prioritize plant core microbiome membership. *Curr Opin Microbiol* 2019;**49**:50–8.

15.Lin H, Peddada S Das. Multi-group Analysis of Compositions of Microbiomes with Covariate Adjustments and Repeated Measures. *Nat Methods* 2024;**21**:83–91.

16.Segata N, Izard J, Waldron L *et al.* Metagenomic biomarker discovery and explanation. *Genome Biol* 2011;**12**

17.Cao Y, Dong Q, Wang D *et al.* microbiomeMarker: an R/Bioconductor package for microbiome marker identification and visualization. *Bioinformatics* 2022;**38**:4027–9.

18.Andrews S, Maria S, Mioreng N *et al.* FastQC: a quality control tool for high throughput sequence data. *Babraham Bioinforma* 2010.

19.Wick RR, Judd LM, Gorrie CL *et al.* Unicycler: Resolving bacterial genome assemblies from short and long sequencing reads. *PLoS Comput Biol.* 2017;**13(6)**:e1005595.

20.Gurevich A, Saveliev V, Vyahhi N *et al.* QUAST: Quality assessment tool for genome assemblies. *Bioinformatics* 2013;**29**:1072–5.

21.Alikhan NF, Petty NK, Ben Zakour NL *et al.* BLAST Ring Image Generator (BRIG): simple prokaryote genome comparisons. *BMC Genomics* 2011;**12**:402.

22.Seemann T. Snippy: fast bacterial variant calling from NGS reads. 2015.

23.Seemann T. Prokka: rapid prokaryotic genome annotation. *Bioinformatics.* 2014;**30**:2068–9.
